# Exploration of Lamiaceae in Cardio Vascular Diseases and Functional Foods: Medicine as Food and Food as Medicine

**DOI:** 10.3389/fphar.2022.894814

**Published:** 2022-06-14

**Authors:** Ishani Chakrabartty, Yugal Kishore Mohanta, Amilia Nongbet, Tapan Kumar Mohanta, Saurov Mahanta, Nibedita Das, Muthupandian Saravanan, Nanaocha Sharma

**Affiliations:** ^1^ Department of Applied Biology, School of Biological Sciences, University of Science and Technology Meghalaya (USTM), Meghalaya, India; ^2^ Department of Botany, School of Biological Sciences, University of Science and Technology Meghalaya (USTM), Meghalaya, India; ^3^ Natural and Medical Sciences Research Centre, University of Nizwa, Nizwa, Oman; ^4^ National Institute of Electronics and Information Technology (NIELIT), Guwahati Centre, Guwahati, India; ^5^ AMR and Nanotherapeutics Laboratory, Department of Pharmacology, Saveetha Dental College, Saveetha Institute of Medical and Technical Sciences, Chennai, India; ^6^ Institute of Bioresources and Sustainable Development, Imphal, India

**Keywords:** Lamiaceae, ethnopharmacology, CVD, functional food, medicine

## Abstract

In the current scenario, cardiovascular disease (CVD) is one of the most life-threatening diseases that has caused high mortality worldwide. Several scientists, researchers, and doctors are now resorting to medicinal plants and their metabolites for the treatment of different diseases, including CVD. The present review focuses on one such family of medicinal plants, called Lamiaceae, which has relieving and preventive action on CVD. Lamiaceae has a cosmopolitan distribution and has great importance in the traditional system of medicine. Lamiaceae members exhibit a wide range of activities like antioxidant, antihyperlipidemic, vasorelaxant, and thrombolytic effect, both *in vitro* and *in vivo*–these are mechanisms that contribute to different aspects of CVD including stroke, heart attack, and others. These plants harbour an array of bioactive compounds like phenolic acids, flavonoids, alkaloids, and other phytochemicals responsible for these actions. The review also highlights that these plants are a rich source of essential nutrients and minerals like omega-3 and hence, can serve as essential sources of functional foods—this can have an additional role in the prevention of CVDs. However, limitations still exist, and extensive research needs to be conducted on the Lamiaceae family in the quest to develop new and effective plant-based drugs and functional foods that can be used to treat and prevent cardiovascular diseases worldwide.

## 1 Introduction

One of the fatal diseases today is cardiovascular disease or CVD. Such diseases include a variety of disorders like stroke, heart failure, myocardial infarction, and hypertension. According to the World Health Organization (WHO), CVD accounts for 80% of death worldwide and is the most rapidly increasing cause of death globally (Gaziano et al., 2010; Rastogi et al., 2016; Chopra et al., 2022). All associated diseases of CVD are characterized mainly by insufficient oxygen supply to the brain and heart. This occurs due to excessive deposition of fats, lipids, and oils in the cerebral and coronary arteries, which lead to the narrowing and subsequent blockage of the pathway for blood flow (Roth et al., 2017). Hypertension, the most common CVD today, has turned fatal due to fast-paced lifestyles, stress, lack of adequate physical activity, and unhealthy food habits (Shaito et al., 2020). Though many drugs like warfarin are used to treat strokes and hemorrhage, they have not been able to lower the death rate due to CVD (Lymperopoulos et al., 2013; Michel et al., 2020). Under such conditions, scientists and chemists need to search for and resort to other suitable alternatives; herbal medicines and plant sources may serve as the possible safe option for treating this deadly disease.

Food and lifestyle are very crucial today for the prevention of diseases and to lead a healthy life. Functional or fortified foods and nutraceuticals are gaining tremendous importance in this regard. According to Healthline, those foods (with low trans-fat) that have health benefits beyond their usual nutritional value, which are rich or fortified with vitamins, minerals, fiber, probiotics, antioxidants, good quality fats, secondary metabolites, and promote growth and development are called functional foods (Linkr, 2020). Such foods can help modulate lipid metabolism, which can prevent fat deposition and obesity; this further contributes to minimizing the risk of CVD (Sikand et al., 2015). Foods rich in secondary metabolites and bioactive compounds like flavonoids, alkaloids, and others are recommended for consumption by dietary guidelines to prevent stress, hypertension, and CVD (Jhonston, 2009; Rivera et al., 2010). Many families of plants like Asteraceae (e.g., *Artemisia campestris* L.), Zingiberaceae (e.g., *Alpinia galanga* (L.) Willd.), Caryophyllaceae (e.g., *Corrigiola litoralis* subsp. foliosa (Pérez Lara) Devesa) and others are very rich in secondary metabolites and have been consumed since time immemorial for their health benefits, without the knowledge of nutraceuticals (Tungmunnithum et al., 2018; Chakrabartty et al., 2020).

Plants form one of the essential footholds of modern drugs (Das et al., 2015; Panda et al., 2019). For many years, traditional medicinal practices like Ayurveda, Unani, and others have been based on plant-based compounds; these practices are still religiously followed in some of the secluded parts of India, China, and other developing nations as a hierarchical legacy (Fabricant and Farnsworth, 2001; Panda et al., 2016). Modern medical research often doubts the authenticity of traditional methods; however, practitioners have not deterred from their path. Most of them try to preserve such age-old knowledge within families. Hence minimal literature or documentation can be found. It is interesting to note that according to WHO, more than 80% of the global population still rely on traditional and herbal medicines, even for life-threatening diseases. Most medicinal plants have the added advantage that they are consumed worldwide as foods. For many years, different species from the Zingiberaceae family are used as traditional medicines as home remedies for the treatment of certain diseases in Southeast Asian countries like India, like decoction of *Alpinia nigra* (Gaertn.) Burtt to treat gut infection by *Fasciolis*, *Curcuma* sp. for wound healing, *Zingiber* sp. for viral infections etc. (Kunnumakkara et al., 2008; Roy et al., 2012; Swargiary et al., 2013; Chakrabartty et al., 2019). Another such family is Lamiaceae, which is very rich in medicinal plants and has long been used as traditional medicine.

Lamiaceae, or mint family, is a widely distributed family of angiosperms that consists of 236 genera with more than 7,000 species (Michel et al., 2020). The largest genera that belong to this family are *Salvia, Scutellaria, Stachys, Ajuga, Plectranthus, Hyptis, Teucrium, Vitex, Thymus, Nepeta*, etc. The different species from this family inhabit diverse ecosystems and have a great diversity with a cosmopolitan distribution. Members of plants this family are important in various industries like perfumery, pharmaceutics, cosmetics, food, fragrance, and others. Such diverse applications lead to the widespread cultivation of the plants of Lamiaceae are, therefore, grown to serve as sources of functional food (Hyde et al., 2014; Li et al., 2016; Zhao et al., 2021). In addition, a number of these plants are aromatic, which can be attributed to a wide variety of complex bioactive compounds that further possess high biological activity both *in vitro* and *in vivo*. Secondary metabolites that show a high amount of potential, like antioxidant, antimicrobial, and anticancer effects, and are vital for their biological efficacy; hence, plants of Lamiaceae play an important role in the treatment of many diseases, including CVD (Stevens, 2012).

The present review aims at bridging the gap in existing literature regarding the role of several members of the family Lamiaceae, particularly in the prevention of CVD and their application as functional foods. Further, it is sub-divided into different sections: Section 2 describes the importance of “mint,” the representative term for Lamiaceae and how it is searched for globally. The following section (Section 3) highlights the role of the Lamiaceae family in the prevention and cure of CVD and its associated diseases. In Section 4, the different bioactive compounds from other species of Lamiaceae are described that have a preventive role in CVD. In Section 5, the role of Lamiaceae as functional foods has been discussed in detail. The challenges and opportunities to utilize the plants of the Lamiaceae family as medicines and nutraceuticals have also been discussed in the subsequent portion (Section 6). In Section 7, the prospects of research in Lamiaceae (in search of novel bioactive compounds to be used as medicines and fortified foods) and the concluding remarks on the present study have been discussed.

Most of the information provided in this manuscript has been collected from freely available journal articles, book chapters, monographs, online articles, etc. that are cited in the text. Electronic databases like Google Scholar, Library Genesis, and Sci-Hub have been used for the data collection. The presently accepted nomenclature of the plants has been used throughout the text and checked for validity. Most of the data referred to here, include the latest and updated information between 2010 and 2022; however, some old data from 2003 to 2009 have also been referred and used because of the relevant information.

## 2 World-Wide Ethnopharmacological Exploration of Lamiaceae

**FIGURE 1:**
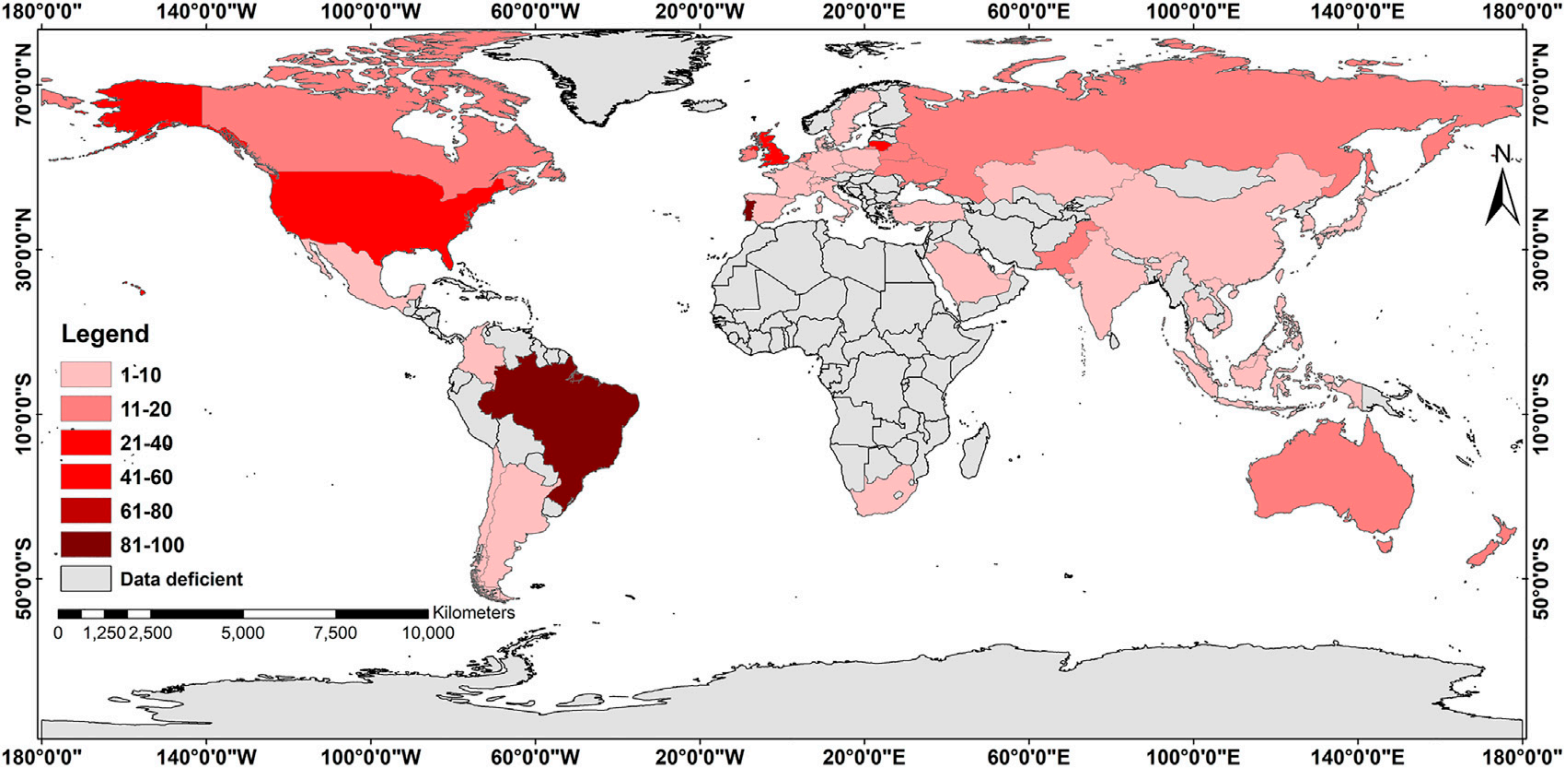
Worldwide exploration of Lamiaceae for ethnopharmacological evaluations and other use. (Figure Source: The Authors and the figure is made using ArcGIS Pro software).

Mint, the refreshing flavour loved by people globally, is associated with the Lamiaceae family. Several people search about mint and its usage in Google to know about it. Google records these search terms and provides a comprehensive idea about the trend of search terms. Therefore, we searched for Google trends (from January 2004 to February 2022) to understand people’s opinions towards the mint and its flavour. Our search criteria ranged Google trend results revealed that the term “mint” was searched for the highest number of times in December 2021 and the lowest in September 2008 (Supplementary Material S1). The global search for the term “mint” is continuously increasing with a substantial increase in the blue colour’s depth is mentioned in the specific territory of different parts of the world (Figure 1). However, in February 2022, it witnessed a downward trend. If country-specific search trends are considered, the people of Brazil have the highest interest in the search term mint followed by Portugal, the United States, and the United Kingdom (Supplementary Material S2).

In contrast, France showed the lowest data in terms of search results. However, at least 205 countries/regions of the globe have little or no interest in mint (Supplementary Material S2). This shows that most of the global population still does not have an interest in mint; it is concentrated in only 45 countries. Though some countries might have searched for mint in Google trends, their search was not enough to make a considerable impact recorded in Google compared to Brazil. Mint is a representative member of Lamiaceae and hence, the term “mint” is believed to represent the entire family. This plant has a characteristic property related to a cooling and stimulating effect in the breath and, later, the entire body. Brazil is a tropical nation that records very high temperatures. This may be one of the probable reasons why the search term “mint” is very commonly googled by the people of this country compared to the cold European nation of France.

## 3 Activity of Lamiaceae in CVD

**FIGURE 2:**
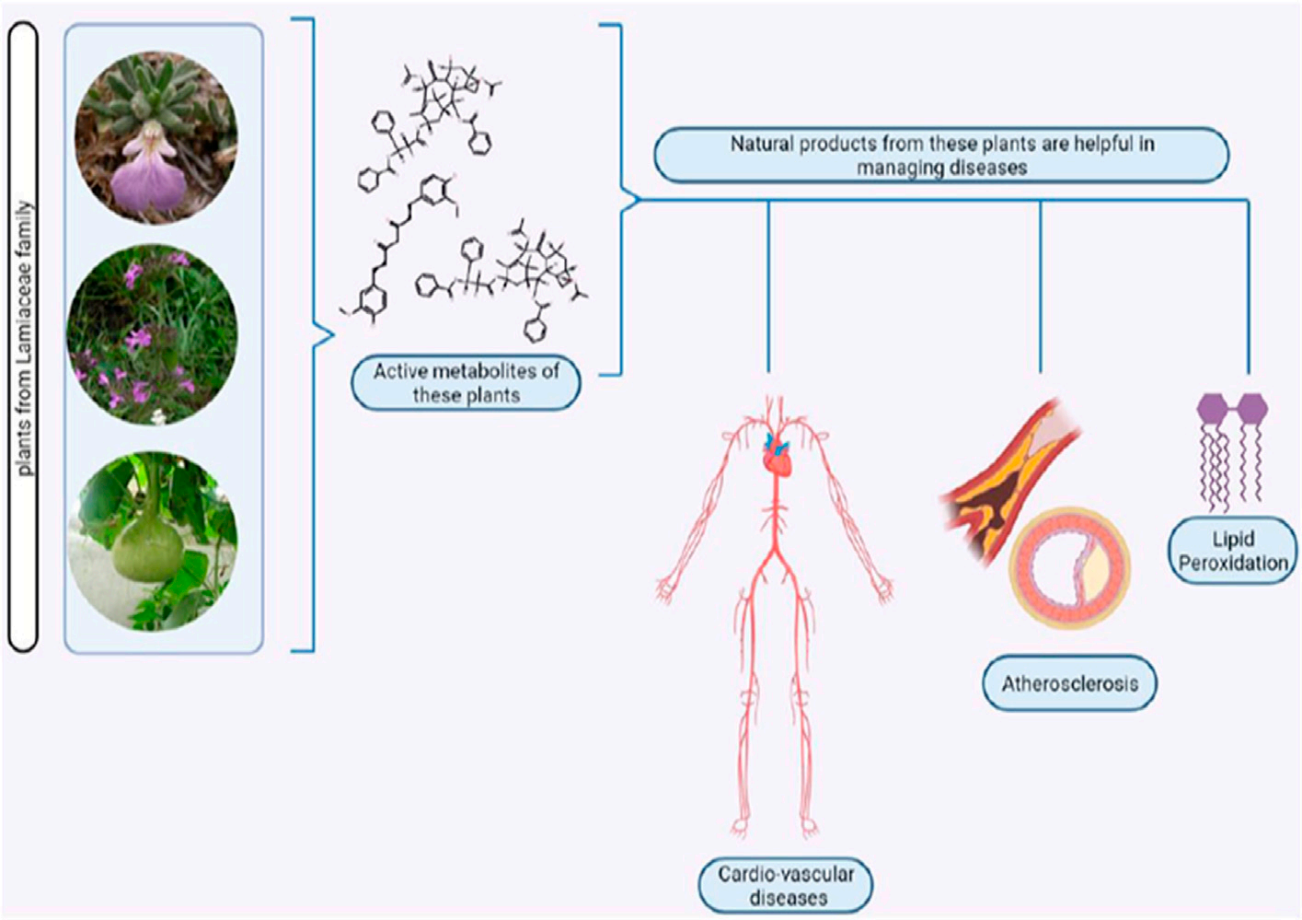
Exploration of Lamiaceae bioactive natural products on CVD (Figure Made in BioRender.com).

A lot of plant species, belonging to different families like Zingiberaceae, Asteraceae, and others, are explored presently that have a wide range of activities like analgesic, anti-inflammatory, antioxidant, vasodilating properties, etc.—many of which can help to prevent CVD (De Souza et al., 2013; Basak et al., 2018; Chakrabartty et al., 2019; Michel et al., 2020). The subsequent sections discuss in detail how different species of Lamiaceae contribute to the prevention of CVD (Table 1, Figure 2); their toxicity profile has also been highlighted (Supplementary Table S1).

**TABLE 1:** Mechanism of action of plants from Lamiaceae in the prevention of CVD.

Sr. No.	Plant name		Plant organ/Extract type		Mechanism of action		References		
1.	*Ajuga iva* (L.) Schreb.		The whole plant (Aqueous extract)		Reduced blood plasma cholesterol and triglyceride levels; lowered lipid peroxidation		Taleb-Senouci et al. (2009)		
2.	*Ajuga integrifolia* Buch. -Ham. ex D. Don		Leaves (methanolic extract)		Caused significant diuresis; aqueous extract showed diuresis at high conc. after 5th hour of administration		Hailu and Engidawork, (2014)		
3.	*Clinopodium vulgare* L.		Aerial parts (Crude and methanolic extract)		Lowered the blood pressure and hypertension (10–30 mg kg^−1^)		Khan et al. (2018)		
4.	*Dracocephalum moldavica* L.		Aerial parts (As food or decoction)		Fraction exhibited high antioxidant and free radical scavenging activity (>70 mg L^−1^)		Jiang et al. (2014)		
5.	*Lavandula stoechas L.*		Aerial parts (Ethanolic extract)		Exhibited very high antioxidant activity (12 μg ml^−1^); value comparable to BHT		Ez Zoubi et al. (2014)		
6.	*Ziziphora clinopodioides* Lam.		The whole plant (Decoction of whole plant)		Acted on voltage-gated K_+_ channels, mobilized Ca^2+^ ions and caused relaxation of vascular smooth muscles		Senejoux et al. (2010)		
7.	*Orthosiphon aristatus* (Blume) Miq.		The whole plant (Aqueous extract)		Caused vasorelaxation effect on endothelium-aortic rings and porphyrin rings by KCl induced mechanism using NO/cGC/cGMP pathways		Yam et al. (2016), Yam et al. (2018)		
8.	*Vitex megapotamica* (Spreng.) Moldenke		Leaves (Aqueous extract)		Lowered plasma cholesterol (500–1,000 mg kg^−1^) and LDL level; prevented formation of atherosclerotic plaques		Pires et al. (2018)		
9.	*Salvia officinalis* L.		Shoots (Crude material, essential oils)		Inhibited lipid peroxidation; had high antioxidant property by scavenging free radicals of oxygen		Dorman et al. (2003), Hossain et al. (2010), Michel et al. (2020)	
10.	*Thymus* *saturejoides* Coss.		The whole plant (extract type unknown)		Significantly lowered plasma levels of triglycerides (conc. 0.2 g 100g^−1^) and cholesterol, along with LDL levels		Ramchoun et al. (2012), Khouya et al. (2015)		
11.	*Salvia miltiorrhiza* Bunge *Salvia miltiorrhiza* var. charbonnelii (H.Lév.) C.Y.Wu		Roots (Oral consumption of dried root)		Inhibited platelet aggregation in the blood and reduced weight of thrombus (clot)		Fan et al. (2010)		
12.	*Lepechinia caulescens* (Ortega) Epling		Aerial parts (Methanolic extract; decoction or tea)		Significantly lowered heart rate and blood pressure; had vasorelaxation effect (conc. 30 and 120 mg kg^−1^)		Estrada-Soto et al. (2012)		
13.	*Prunella vulgaris* L.		The whole plant (Hydroacoholic and aqueous extract)		Increased HDL concentration in the blood; exhibited free radical scavenging activity on superoxide and hydrogen peroxide.		Zargar et al. (2017), Michel et al. (2020)		
14.	*Pogostemon elsholtzioides* Benth.		Leaves (Essential oil; leaf decoction)		Caused relaxation of contracted aortic rings in a dose-dependent manner and lowered heart rate		Shiva Kumar et al. (2017)		
15.	*Leucas aspera* (Willd.) Link		Leaves (Ethanolic extract)		Lowered serum levels of cholesterol and triglycerides (conc. 100–200 mg kg^−1^)		Vijay Kumar and Devanna, (2016)		
16.	*Leonurus cardiaca* L.		Aerial parts (Refined extract given as infusion)		Reduced pressure on the ventricles and eased flow of blood through coronary arteries in a dose-dependent manner (conc. 1–2 mg ml^−1^)		Ritter et al. (2010)		
17.	*Sideritis raeseri* Boiss. & Heldr.		Aerial parts (Extract type unknown)		Decreased blood pressure and heart rate in a dose-dependent manner, and led to vasodilation		Kitic et al. (2012)		
18.	*Satureja cuneifolia* Ten. (syn.		The whole plant (Aqueous extract)		Inhibited KCl and adrenaline-induced contraction of smooth muscles at toric phase in a concentration-dependent manner		Ramón Sánchez de Rojas et al. (1999)		
19.	*Teucrium polium* L.		Aerial parts (Aqueous extract)		Lowered levels of cholesterol and triglycerides in the blood in a dose-dependent manner; exhibited diuretic effect		Michel et al. (2020), Rasekh et al. (2001)		

^a^All the scientific names of the plants are mentioned in the table according to version 1.1 of the Plant List Published on the Internet (The Plant List, 2013) and http://mpns.kew.org/mpns-portal/.

### 3.1 Antioxidant Activity

Antioxidants are those chemical substances that can negate the harmful effects of free radicals and hence prevent the tissues from getting damaged. As such, they help safeguard against CVD (Michel et al., 2020). The generation of reactive oxidative species (ROS) can lead to oxidative stress and attack the vital biomolecules of the body, leading to damage to endothelial cells and vascular tissues. This can also lead to atherosclerosis and the conditions may become fatal over time (Taleb et al., 2018). It has been reported that natural antioxidants, especially from plant sources, have a tremendous potential for scavenging free radicals, chelating metal ions, enhancing the endogenous antioxidant system, and further preventing the formation of reactive species (Supplementary Table S3); hence, there are an intense and aggressive search plant-based antioxidants (Amic et al., 2003; Seyoum et al., 2006). Common bioactive drugs of Lamiaceae like oregano (*Origanum vulgaris* L.), rosemary (*Rosemarinus officinalis* L.), basil (*Ocimum basilicum* L.), thyme (*Thymus vulgaris* L.), and sage (*Salvia officinalis* L.) possess high antioxidant properties; they are reported to have free radical scavenging activity which increases upon heat exposure and drying (Hossain et al., 2010). Medicinal plants of Lamiaceae, *Lycopus europaeus* L., *Melissa officinalis* L., and *Prunella vulgaris* L. have shown significant anti-oxidative activities by free radical scavenger effect on DPPH (SC_50_ values ranging from 16.7 ± 1.1 to 221.8 ± 49.0 µΜ for the three plants), which have been reported to be higher than that of rosmarinic acid (SC_50_: 5.5 ± 0.2 µM) (Michel et al., 2020). *Thymus sipyleus* Boiss. subsp. *sipyleus* var. *sipyleus*, *Teucrium chamaedrys* L., *Mentha longifolia* (L.) Hudson subsp. *longifolia*, *Salvia limbata* C.A. Meyer, and *Thymus fallax* Fisch. and Mey, are very rich in phenols and flavonoids—these impart antioxidant properties to the extracts of the plants which are capable of regenerating antioxidant properties in the lipid bilayer of cells (Özgen et al., 2006). Firuzi et al. (2010) estimated the antioxidant activity of 24 species of Lamiaceae from Iran that included plants like *Ballota aucheri* Boiss., *Eremostachys adenantha* Jaub. & Spach Ill*, Otostegia michauxii* Briq.*, Phlomis elliptica* Benth.*, Phlomis olivieri* Benth.*, Phlomis persica* Benth.*, Thymus daenensis* Celak*.*, and nine species of *Salvia* by DPPH and FRAP assay—of these, *Salvia santolinifolia* Boiss. has the highest antioxidant activity, comparable to the positive control, ascorbic acid (Firuzi et al., 2010; Michel et al., 2020). The essential oils were obtained from the aerial parts of *Lavandula angustifolia* Mill. and *Lavandula stoechas* L. also show high antioxidant activity (Ziaee et al., 2015). In addition, the ethanolic extract of the leaves of sage contains a high amount of phenolic acids, which exhibit high antioxidant activity as the phenol ring can easily give up H_2_ atoms (Ramu et al., 2012).

### 3.2 Blood Cholesterol and Lipid-Lowering Activity

An increase in blood cholesterol and triglycerides are another cause of CVD. It can lead to atherosclerosis—the condition in which fat droplets and lipids get deposited and hardened in the arteries obstructing the free flow of blood. This leads to various cardiac and coronary diseases like stroke and vascular diseases. The ability of substances to break down these hardened fat depositions is called the antihyperlipidemic or hypolipidemic effect, which can lower the level of triglycerides like low-density lipoprotein (LDL) and cholesterol and help to increase the “good” high-density lipoprotein (HDL) (Akinpelu et al., 2016). Thus, they can alleviate the risk of CVD greatly. The extracts obtained from the different plant organs of Lamiaceae are very rich in polyphenols and exhibit an antihyperlipidemic effect. The dried hydroalcoholic extract *Dracocephalum kotschyi* Boiss (dosage 40 mg kg^−1^ body weight) has been reported as the best dose for hyperlipidemia in rats and can significantly reduce the risk of atherosclerosis (Heydari et al., 2019). The ethanolic and ether extracts of *Leucas aspera*, which are used in folklore to treat various ailments, are rich in secondary metabolites. The steroidal phytochemicals tend to replace cholesterol in triglyceride formation that further leading to a hypolipidemic effect; the extract was effective in a dose-dependent manner in rat modes with induced hyperlipidemia (dexamethasone treatment) (Vijay Kumar and Devanna, 2016). Indian tulsi (*Ocimum tenuiflorum* L.) is traditionally used as an anti-tussive agent. The alcoholic extract of this plant has a no. of phenolic compounds—each of which has anti-hyperlipidemic activity. In addition, the extract (125–500 mg kg^−1^ body weight) can lower the level of LDL and increase HDL up to the story of a healthy rat (Parasuraman et al., 2015). Aqueous extracts of *Thymus saturejoides* Coss. (0.2 g per 100 g of extract) can significantly lower blood plasma cholesterol and triglyceride levels—this decrease is associated with the reduction of LDL levels. Similarly, the aqueous extract of *Teucrium polium* L. (50–150 mg kg^−1^ of extract) can decrease levels of cholesterol and triglycerides in the blood of hyperlipidemic rats in a dose-dependent manner (Khouya et al., 2015).

### 3.3 Anticoagulation and Thrombolytic Activity

Blood clotting involves a complex series of events—the breakdown to protein activates the process of thrombin formation that further converts fibrinogen to fibrin. Factor VIII with platelets triggers this set of circumstances and aid in the process of blood clotting or blood coagulation. The formation of a thrombus or clot in the arteries and tissues is prevented by anticoagulants (Schaeffer and Zimlich, 2019). Few plants of Lamiaceae have been reported to possess anticoagulation potential. Ali et al. (2014) revealed thrombolytic activity of methanol extract and solvent fractions (petroleum ether, chloroform, carbon tetrachloride) and aqueous fractions of the leaves of *Clerodendrum infortunatum* L. by clot lysis assay; the carbon tetrachloride and chloroform fractions displayed the highest and least percentage of clot lysis. The different bits of the root extract of this plant also showed thrombolytic activity (Ali et al., 2014; Prashith Kekuda et al., 2019). The leaf extracts of *Salvia officinalis* L., *Thymus vulgaris* L. and *Rosmarinus officinalis* L. (200–1,000 μg ml^−1^) exhibit thrombolytic activity in a dose-dependent manner; their thrombolytic potential can be arranged in the order *Salvia officinalis* L. > *Thymus vulgaris* L. > *Rosmarinus officinalis* L. (Mutalib, 2015)*.* In addition, *Salvia miltiorrhiza* (Bunge) also exhibited mild inhibition of platelet aggregation *in vivo* in rats. Another member of Lamiaceae, *Leonotis leonurus* (L.) R. showed anticoagulation potential both *in vitro* and *in vivo*. The plant extract lowered or prevented the expression of proteins responsible for platelet aggregation and blood clotting. At a concentration of 50–100 μg ml^−1^, platelet adhesion was reduced in a dose-dependent manner, and preventing activation of thrombin and fibrinogen (Mnonopi et al., 2011).

### 3.4 Inhibitory Action on Hypertension

Excessive contraction of blood vessels for a prolonged duration causes an increase in blood pressure, leading to hypertension. Vasorelaxants are drugs that can cause dilation of blood vessels that ensure the flow of blood through the vessels at ease—most of these cause relaxation of vascular smooth muscles—the most common method of treatment used for hypertension (Herradón et al., 2007). It has been reported that plant-based compounds like chalcones inhibit angiotensin-converting exam (ACE) through nitric oxide (NO)- and estrogen receptor α (ERα)-dependent pathways to bring about vasorelaxation and lower the risk of CVDs (Legeay et al., 2020). *Agastache mexicana* (Kunth.) Lint. and Epling, is a medicinal plant from the Lamiaceae family used for the treatment of anxiety. The antihypertensive activity of the plant extract was determined in the male rat - the extract exhibited relaxant activity and exhibited vasodilation effect through several receptors, such as the augment of free cytosolic Ca^2+^ levels; it further inhibited vasoconstriction and showed a diuretic effect, preventing hypertension (Hernandez-Abreu et al., 2013; Flores-flores et al., 2016). Senejoux et al. (2010) reported the vasodilation activity of *Ziziphora clinopodioides* Lam., a plant that finds its use in Chinese folk medicine for the treatment of hypertension. The decoctions of the whole plant of *Z. clinopodioides* decreased the influx of Ca^2+^ ions in KCl mediated contractions of vascular smooth muscles. Further, the vasorelaxation of this extract did not involve endothelium-derived relaxing factors like NO and prostacyclin (Senejoux et al., 2010). The sections of *Clinopodium vulgare* L., another member of the mint family, have vasodilation potential in rats both *in vitro* and *in vivo*. At a concentration of 1–30 mg kg^−1^, the vasorelaxation in rat aorta was endothelium mediated, *i.e.* it involves muscular NO channels, K^+^ channels, together with the closure of Ca^2+^ ion channels, and occurred in a dose-dependent manner (Khan et al., 2018). The extract of *Orthosiphon aristatus* (Blume) Miq. Possesses vasorelaxation or vasodilation activity, which exhibited a relaxation on KCl-induced aortic rings of the endothelium and phenylephrine-induced aortic ring that may or may not have endothelium in rats; pathways that are involved in this vasorelaxant activity are NO/sGC/cGMP pathways (Yam et al., 2016, 2018). Other members of Lamiaceae that are engaged in vasorelaxation and vasodilation are *Melissa officinalis* L., *Orthosiphon aristatus* (Blume) Miq. (syn. *Orthosiphon stamineus* Benth.), *Phlomoides bracteosa* (Royle ex Benth.) Kamelin and Makhm., *Plectranthus hadiensis* (Forssk.) Schweinf. ex Sprenger (syn. *Coleus forskohlii* Willd.), *Pogostemon elsholtzioides* Benth, *Satureja cuneifolia* Ten. (syn. *Satureja obovata* Lag.), *Sideritis raeseri* Boiss. and Heldr (Michel et al., 2020).

### 3.5 Diuretic Activity

Drugs that aid in diuresis can increase the urinary volume; this can lower the risk of heart disorders and ease out conditions like heart failure, pulmonary oedema, and stroke. Many drugs are available that can be used alone or in combination for diuresis, but they have a myriad of adverse effects with them. Research on plants and plant-based bioactive compounds suggests that plant species exhibit diuretic activity with negligible side effects; some plants of the Lamiaceae family have been reported for the same (Gupta and Neyses, 2005; Morganti, 2005; Michel et al., 2020). The different solvent extracts (aqueous, alcoholic and ethyl acetate) of the leaf of *Plectranthus amboinicus* (Lour) Spreng increases urine volume and decreases serum Na level of albino rats in comparison to the available drug but had no effect on the serum K level; the ethyl acetate fraction was more potent as a diuretic group with better electrolyte balance than the other solvent fractions (El-Hawary et al., 2012). The aqueous and ethanol extracts of *Coleus amboinicus*, Lour. Has also shown significant diuretic activity (Venkateshappa and Sreenath, 2013). *Ajuga integrifolia* Buch. -Ham. ex D. Don) is a perennial herbaceous plant of Lamiaceae that has significant diuretic activity; its aqueous and 80% methanolic extract showed an increase in urine volume and the diuretic effect, together with electrolyte excretion effect, was comparable to that produced by diuretic drug furosemide (Hailu and Engidawork, 2014). *Clerodendrum myricoides* Hoscht. has been used as a traditional medicine for curing various ailments like urine retention and oedema. This plant’s hydromethanolic leaf and root extracts and its fractions (ethyl acetate, chloroform, and alcohol) showed diuretic activity in rats *in vivo*—the hydromethanolic extract showed higher diuresis than furosemide (100 mg kg^−1^). It is reported that this extract had higher solubility for pharmacologically active ingredients like flavonoids, tannins, terpenes, phenols, saponins and others, and they also act synergistically; moreover, this extract has very minimal cell cytotoxicity (Welu et al., 2020). Other plants from Lamiaceae that show diuretic activity are *Anisomeles indica* L., *Teucrium polium* L., and *Ajuga integrifolia* Buch. -Ham. ex D. Don (Malki and Yahia, 2014)*.*

## 4 Bioactive Compounds From Lamiaceae and Their Role in CVD

According to the National Cancer Institute (NCI) dictionary, a bioactive compound is a chemical compound that is present in minimal amounts in living systems like plants and microorganisms, and even foods like nuts and cereals. These compounds have benefits to human health like promoting growth, antimicrobial potential, and prevention of diseases like cancer and CVD (https://www.cancer.gov/publications/dictionaries/cancer-terms/def/bioactive-compound). Most families of plants like Euphorbiaceae, Zingiberaceae, Asteraceae, and others possess a large no. of bioactive compounds that have different medicinal properties and mainly include secondary plant metabolites like flavonoids, terpenoids, chalcones, alkaloids, saponins, tannins etc. It is important to note that the quantity of bioactive compounds produced by any plant has a powerful influence on the environment. For instance, the essential oil content from the leaves of *Alpinia nigra* (Gaertn.) Burtt is slightly higher in Assam than in Bangladesh. The climate, temperature, soil, harvest time, other ecological factors, and the mode of oil extraction used, may influence the oil yield (Ghosh et al., 2013; Islam et al., 2014).

**FIGURE 3:**
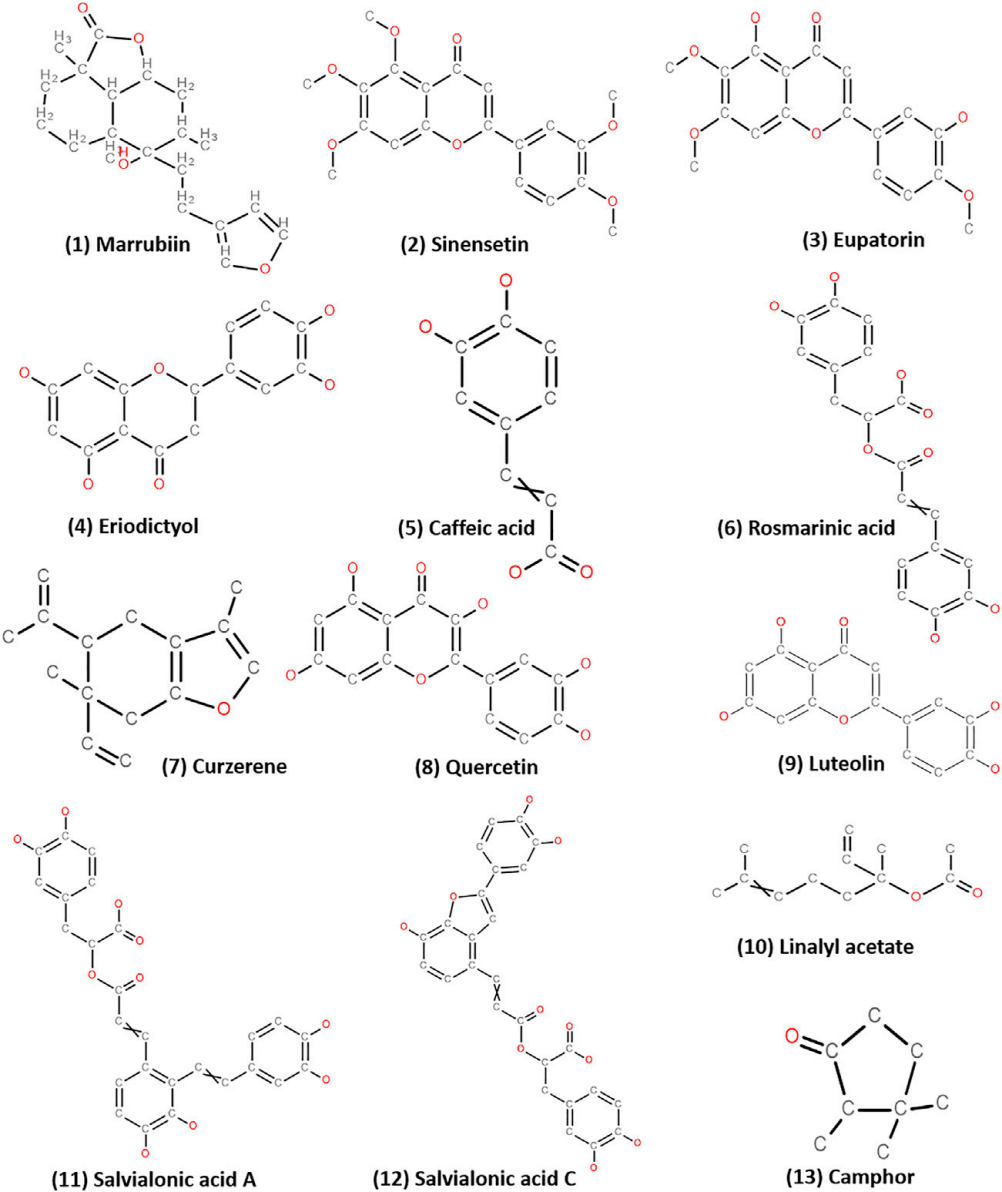
Bioactive compounds from Lamiaceae that can be derived as potential lead drugs for the treatment of CVDs (Biovia software was used to prepare chemical structures https://www.3ds.com/products-services/biovia/products/scientific-informatics/biovia-draw/).

**FIGURE 4:**
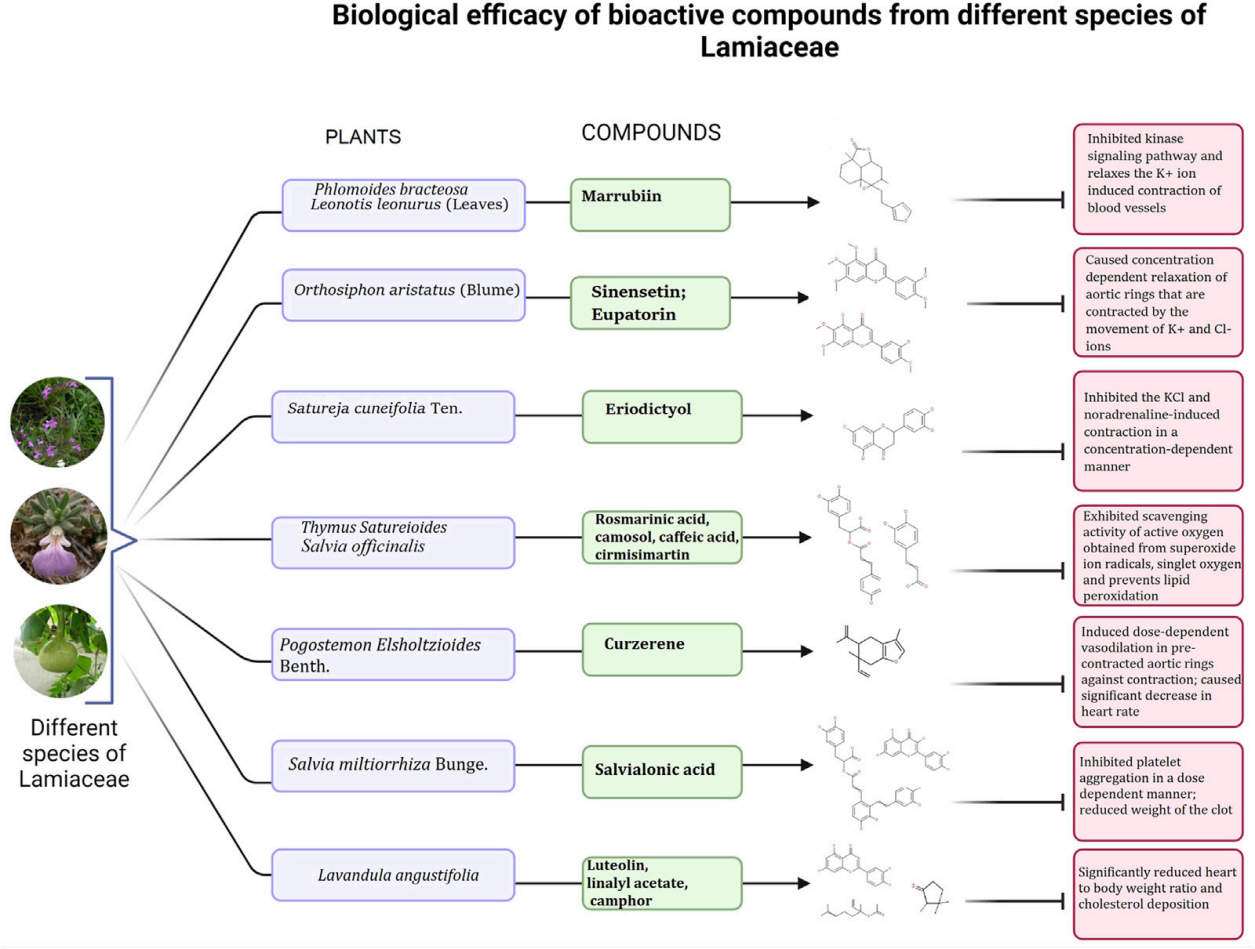
Schematic representation of the mechanism of action of the bioactive compounds of Lamiaceae in the treatment of CVDs (Figure made in Microsoft paint).

Many plants of Lamiaceae like *Micromeria macrosiphon* Coss., *Plectranthus monostachyus* (P. Beauv.) B.J. Pollard, *Ballota glandulosissima* Hub. -Mor. & Patzak, *Lallemantia royleana* (Benth.) Benth., *Thymus dreatensis* Batt. and many others play an important role in preventing CVD and its associated disorders; such potential is also imparted to the plant by the presence of pharmacologically functional elements or bioactive compounds present in the different plant parts at varying concentrations (Table 2). A number these compounds can be derived as potential lead drugs for the treatment of CVDs (Figure 3); their mechanism of action has also been provided (Figure 4; Table 2). These compounds have been isolated primarily by column chromatography and characterized extensively using several analytical techniques like preparative Thin Layer Chromatography (pTLC), High-Performance Liquid Chromatography (HPLC), Ultraviolet (UV) spectral analysis, Fourier Transform Infrared Spectroscopy (FTIR), 13C and 1H Nuclear Magnetic Resonance (NMR) and Mass Spectroscopy (MS) (Ghosh and Rangan, 2013; Chakrabartty et al., 2018). The clinical trial data of these compounds have also been included (Supplementary Table S2). A bioactive compound, marrubiin, isolated from *Leonotis leonurus* (L.) R. Br. can prevent platelet aggregation and lyse thrombus both *in vitro* and *in vivo.* This diterpenoid also lowers insulin secretion, LDL and blood cholesterol, IL-1β and IL-6 levels, and increases HDL concentration (Mnonopi et al., 2011; Mazimba, 2015). Indian tulsi has a number of bioactive compounds like eugenol, rosmarinic acid, carvacrol, linalool, estragole, caryophyllene, ursolic acid, apigenin, and cirsimaritin, which have antihyperlipidemic activity (Pattanayak et al., 2010; Jamshidi and Cohen, 2017). *Origanum vulgare* L. or Oregano is rich in phenolic compounds with very high antioxidant activity. These phenolic compounds are apigenin, luteolin, rosmarinic acid, 2,5-dihydroxybezoic acid, 3,4-dihydrobezoic acid, caffeic acid, maltol, quercetin, scutellarin and others; most of them have associated biological activities like antiviral, antimicrobial, anti-plasmodial, anticancer etc. It is reported that the phenolic structure of these compounds is responsible for the free radical scavenging activity (Zhang et al., 2014; Gutiérrez-Grijalva et al., 2018). Similarly, the ethanolic extracts of sage (*Salvia officinalis* L.) is rich in flavonoids and phenols like cirsimaritin, apigenin, epirosmanol, hespertin, carnosol; the high antioxidant activity is due to the presence of vast amounts of rosmarinic acid and chlorogenic acid which can donate H-atoms to the free radicals (Generalić et al., 2012; Ramu et al., 2012). Sinensetin (0.262 μg ml^−1^) and eupatorin, bioactive compounds from *Orthosiphon aristatus* (Blume) Miq. have vasorelaxant activity that utilizes the NO/cGC/cGMP pathways. In addition, they have anticancer solid potential and other pharmacological properties, with minimal or no toxicity to the target tissues (Yam et al., 2018; Han Jie et al., 2021). The methanolic extract of purple Himalayan mint or *Phlomoides bracteosa* (Royle ex Benth.) Kamelin & Makhm. contains two compounds marrubiin and phlomeoic acid, which have vasorelaxation properties—marrubiin can block the voltage-gated channel of Ca^2+^ ions and inhibit K^+^ ion mediated contraction of the aortic rings (Khan et al., 2012). Salvianolic acid is an active component obtained from *Salvia miltiorrhiza* (Bunge) that has thrombolytic activity at a concentration of 2–10 mg kg^−1^ in a dose-dependent manner; the compound prevents platelet aggregation by binding with thrombin and, thus, interferes with the clotting signaling pathway (Fan et al., 2010; Michel et al., 2020). Therefore, the Lamiaceae members include a vast number of active ingredients that have a direct or direct role in preventing CVD.

**TABLE 2:** Biological efficacy of bioactive compounds from different species of Lamiaceae.

Sr. No.	Bioactive compound	Plant name	Class of compound/Plant part	Mechanism of action	References
1	Marrubiin	*Leonotis leonurus* (L.) R.Br.	Diterpenoid (Leaves)	Inhibited kinase signalling pathway and relaxes the K+ ion induced contraction of blood vessels	Mnonopi et al. (2011), Mazimba, (2015)
2	Sinensetin and Eupatorin	*Orthosiphon aristatus* (Blume) Miq.	Flavonoid (Whole plant; leaves)	Caused concentration dependent relaxation of aortic rings that are contracted by the movement of K^+^ and Cl^−^ ions	Yam et al. (2018), Han Jie et al. (2021)
3	Eriodictyol	*Satureja cuneifolia* Ten.	Flavone (Whole plant)	Inhibited the KCl and noradrenaline-induced contraction in a concentration-dependent manner	Ramón Sánchez de Rojas et al. (1999)
4	Rosmarinic acid, camosol, caffeic acid, cirmisimartin	*Salvia officinalis* L.	Phenols and flavonoids (Shoots)	Exhibited scavenging activity of active oxygen obtained from superoxide ion radicals, singlet oxygen and prevents lipid peroxidation	Dorman et al. (2003), Hossain et al. (2010), Michel et al. (2020)
5	Curzerene	*Pogostemon elsholtzioides* Benth.	Sesquiterpene (Leaves)	Induced dose-dependent vasodilation in pre-contracted aortic rings against contraction; caused significant decrease in heart rate	Shiva Kumar et al. (2017)
6	Salvialonic acid	*Salvia miltiorrhiza* Bunge	Phenolic acid (Roots)	Inhibited platelet aggregation in a dose dependent manner; reduced weight of the clot	Fan et al. (2010)
7	Caffeic acid, rosmarinic acid, quercetin	*Thymus satureioides* Coss.	Phenolic acid and polyphenol (Whole plant)	Caused significant lowering of plasma triglycerides and blood cholesterol level within 24 h s of administration	Ramchoun et al. (2012), Khouya et al. (2015)
8	Marrubiin and phlomeoic acid	*Phlomoides bracteosa* (Royle ex Benth.) Kamelin and Makhm.	Diterpenoid and phenolic acid (Whole plant)	Blocked voltage-gated channel of Ca^2+^ ions and inhibit K^+^ ion mediated contraction of the aortic rings, leading to relaxation	Khan et al. (2012)
9	Rosmarinic acid, caffeic acid	*Thymus zygis* L.	Phenolic acid (Whole plant)	Prolonged the clotting time and process and inhibited plasma clot formation	Khouya et al. (2015)
10	Luteolin and linalyl acetate camphor	*Lavandula angustifolia* Mill.	Flavonoid and monoterpenoid (Shoots and leaves)	Significantly reduced heart to body weight ratio and cholesterol deposition	Ziaee et al. (2015)

^a^All the scientific names of the plants are mentioned in the table according to version 1.1 of the Plant List Published on the Internet (The Plant List, 2013) and http://mpns.kew.org/mpns-portal/.

## 5 Lamiaceae as Functional Foods

Lamiaceae is an essential group of medicinal plants, comprising mainly aromatic plants containing compounds such as flavonoids, terpenes, phenolic compounds, polyphenols, iridoids, and many other active compounds. The number of Lamiaceae plants used in essential oil production is more than 30 species worldwide that are used as food or food additives to prevent the risk of cardiovascular diseases, cancer, and diabetes. Effective use of Lamiaceae plants is underway to develop new natural products that can help the patients prevent and treat many cardiovascular and other related diseases. Cardiovascular diseases (CVDs) are disorders of the heart and blood vessels with coronary heart disease, heart failure, hypertension, hyperlipidaemia, and thromboembolism. The estimated mortality rate from CVDs as per the World Health Organization (WHO) is 32% of all global deaths. It is expected to reach 23.3 million before 2030 (Suroowan and Mahomoodally, 2015). Medicinal plants have been traditionally used from time immemorial to treat many diseases, including CVDs and related complications. Many of the commercially available drugs for the treatment of CVDs are derived from herbal plants. Lamiaceae family has potent cardioprotective effects as reported by many studies on medicinal and aromatic bioactive drugs (Patrignani et al., 2021). The plants are either used as crude extracts, essential oils (Eos), or by extraction of the active compounds against CVD. The active compounds extracted from this plant family have been reported as promising cardioprotective activity *in vitro* and *in vivo* (Table 3).

**TABLE 3:** List of Lamiaceae species derived functional foods as cardio protective.

Sl.No.	Plant scientific name	Plant organ(s)	Cardioprotective effects and traditional use	References(s)
1	*Salvia miltiorrhiza* Bunge	Roots, aerial parts	Cardiovascular effect, Hypertension; prevention of LDL-C oxidation	Chen et al. (2001), Feng Chen et al. (2017), Wang et al. (2013)
2	*Salvia columbariae* Benth.	Roots	Treatment of stroke and heart attack	Adams et al. (2005)
3	*Thymus vulgaris* L.	Aerial parts	Cardioprotective activity	Mărculescu et al. (2007)
4	*Salvia fruticosa* Mill.	Whole Plant	Used for curing headaches, abdominal pains, indigestion, and heart disorders	Itani et al. (2008)
5	*Ocimum gratissimum* L.	Leaves	Cardioprotective effect	Prabhu et al. (2009)
6	*Salvia officinalis* L.	Leaves	Associated with cardiovascular complications and diabetes	Sá et al. (2009)
7	*Plectranthus barbatus* Andrews	Leaves	Cardioprotective activity	Alasbahi and Melzig, (2010)
8	*Clinopodium umbrosum* (M.Bieb.) K.Koch	Whole Plant	Heart tonic	Rana Man and Samant, (2011)
9	*Satureja montana* L.	Aerial parts	Cardioprotective activity	Wang et al. (2011), López-Cobo et al. (2015)
10	*Scutellaria baicalensis* Georgi.	Aerial parts	Cardiac protection against ischemic heart disease	Wang et al. (2011)
11	*Ocimum basilicum* L.	Aerial parts	Protect the myocardium against isoproterenol induced infarction Anticoagulant effect; Cardioprotective effect of rosmarinic acid	Fathiazad et al. (2012), Pour et al. (2016), Koshma et al. (2018)
12	*Salvia hispanica* L.		Prevent cardiovascular diseases and cardioprotective effects	Muñoz et al. (2013), Karbowska and Kochan, (2018)
13	*Dracocephalum moldavica* L.	Whole plant	cardioprotective effects	Xu et al. (2018)
14	*Mentha arvensis* L.	Fresh leaves	Beneficial effects of M. arvensis in patients with ischemic heart disease	Gul et al. (2014)
15	*Satureja hortensis* L.	Grounded dried leaves	Cardioprotective activity	Damašius et al. (2014)
16	*Lavandula anguistifolia* Mill.	Dried aerial parts	Protects myocardium against isoproterenol induced myocardial infarction that it could be related to its antioxidant properties	Ziaee et al. (2015)
17	*Mentha pulegium* L.	Aerial parts	Cardioprotective effect	Taamalli et al. (2015)
18	*Origanum majorana* L.	Aerial parts	Cardioprotective effect	Taamalli et al. (2015)
19	*Marrubium vulgare* L.	Aerial parts	Protective effect against cardiac complications	Garjani et al. (2017)
20	*Melissa officinalis* L.	Aerial parts	Cardioprotective effect	Skendi et al. (2020b)
21	*Origanum vulgare* L.	Aerial parts, dried, grounded leaves and flowers	Contain cardioprotective, flavonoids	Tzima et al. (2018)

^a^All the scientific names of the plants are mentioned in the table according to version 1.1 of the Plant List Published on the Internet (The Plant List, 2013) and http://mpns.kew.org/mpns-portal/.

### 5.1 Effects of Lamiaceae Functional Food Components as Cardioprotective

Plants of Lamiaceae possess promising benefits in reducing the risk of CVDs through the suppression of inflammation (Tzima et al., 2018). The methanolic extract of *Satureja hortensis* L. plant is well known for CVDs treatment and other related complications by inhibiting the secretion, aggregation and adhesion of the blood platelet and the anticoagulant activity blood (Hamidpour et al., 2014). The leaf extract of *Ocimum basilicum* L. showed an anticoagulant effect, as reported by Pour et al. (2016) (Pour et al., 2016). The *Lavandula aguistifolia* Mill. contain bioactive compounds such as flavonoids, tannins, triterpenoids, coumarins, cineole, limonene, and camphor showed substantial antioxidant properties with reduced cardiac tissue damage and strengthened myocardial membrane (Koshma et al., 2018). The plant extract of *Leonotis leonurus* (L.) R.Br. contains a primary compound named Marrubiin (labdane diterpenoid) exhibiting antiplatelet, anticoagulant, and anti-inflammatory properties (Mnonopi et al., 2011). The *Leonurus turkestanicus* V.I.Krecz. & Kuprian. as reported by (Mamadalieva et al., 2017), shown to be effective against cardiovascular, stomach, and other related diseases. As shown by histopathological analysis, *Stachys schimperi* Vatke possesses cardioprotective effects on DOX-induced cardiotoxicity in rats. Rosmarinic acid found in basil leaves (*Ocimum basilicum* L.) exhibited cardioprotective effects in rats with isoproterenol-induced myocardial infarction (Koshma et al., 2018). One of the major components found in most species of the Lamiaceae family, Thymoquinone (2-isopropyl-5-methylbenzo-1,4-quinone) showed healing effects against apoptosis, coronary artery diseases, diabetes, urinary system failures, hypertension, inflammation, and oxidative stress which is related to its antioxidant and anti-inflammatory activity (Farkhondeh et al., 2017). Components such as methylripariochromene A, orthochromene A, neoorthosiphol A and B and tetramethylscutell isolated from the leaves of *Orthosiphon* species of Lamiaceae family have been studied and showed decreased systolic blood pressure (Singh et al., 2015). Essential oil of *Lavandula angustifolia* Mill. has been shown to protect isoproterenol-induced myocardial infarction in a study using rat antioxidant activity (Ziaee et al., 2015) (Table 3).

Incorporating bioactive drugs from the Lamiaceae family (sage, mint, rosemary, oregano, thyme) into the innovative functional foods on aroma compound formation and sensory properties oat-buckwheat products. Herbs of Lamiaceae contain a unique, complex mixture of bioactive compounds. They include secondary metabolites such as phenolic compounds, tannins, quinines, lignans, terpenoids, and flavonoids and they are also rich in essential oils and can enhance the taste and aroma of the final product (Vaishali Rai et al., 2013; Carović-Stanko et al., 2016; El-Sayed and Youssef, 2019). Most herbs and spices have relatively high micronutrients (minerals and vitamins), macronutrients (such as protein, fat, and carbohydrate) and fewer anti-nutritional properties. The total antioxidant potential of plant materials such as culinary herbs, spices, vegetables, as well as fruits and oilseed products are related to ascorbic acid (vitamin C), alpha-tocopherol (Vitamin E), beta-carotene (Vitamin A precursor), numerous flavonoids, and other phenolic compounds (Rather et al., 2016). Basil (*Ocimum basilicum* L.) is one of the most popular cultivated plants. It is a good source of natural antioxidants and contains significant amounts of essential phytochemicals. The inclusion of basil in the development of fresh cheese made with organic buffalo milk did not modify the fat, protein, moisture, and mineral content. In contrast, the total polyphenol content and antioxidant activity of cheeses increased. It also changed the hardened and chewiness but not influenced springiness and cohesiveness. Thus, the basil improves the functional and modified technological characteristics of fresh cheeses and presents good acceptability (Ribas et al., 2019). Food can be functional by increasing concentration, adding or improving the bioavailability of bioactive ingredients, such as probiotics, fibres, phytochemicals, vitamins, minerals, bioactive drugs, omega-3s, peptides-proteins (Roberfroid, 2002; Arvanitoyannis and Van Houwelingen-Koukaliaroglou, 2005). Basil (*Ocimum basilicum* L.) is rich in polyphenols, antioxidant, antimicrobial and antifungal properties (Carocho et al., 2016). The basil branches mainly contain fibers, essential oils and minerals, such as nitrogen, calcium, potassium and magnesium. Basil leaves have significant amounts of carotene, vitamin B (1, 2, 3), vitamin C, minerals (calcium, phosphorus and iron), polyphenols and essential oils (Dumbrava et al., 2012). Thus, it can be added to foods as a functional ingredient, such as dairy products. Phenolic compounds, secondary outcomes of plant metabolism, have been suggested as bioactive compounds due to their antioxidant capacity and beneficial effects on human health (Han et al., 2011). Additions of seasoning plants, rich in phenolic compounds, in cheeses have already been reported (Asensio et al., 2015).

### 5.2 Active Food Components With Cardiovascular Effect

Some of the bioactive compounds from the Lamiaceae plant family with cardio-protective and therapeutic properties include leonurine, rosmarinic acid, quercetin, apigenin, carvacrol, thymoquinone, baicalein, and many others (Table 3). The cardioprotective effects exhibited by these compounds are through multiple regulations, including growth factors, enzymes, kinases, apoptotic, transcription factors, and other molecules.

Apigenin has shown similar mechanisms in cardio-protection by inhibiting the phosphorylation of p38 MAPKS during myocardial I/R and reducing the activity of caspase-3 activity and Bax protein expression while the expression of Bcl-2 protein is increased (Hu et al., 2015; Chen Y. et al., 2017). Leonurine, a natural active compound of *Leonurus cardiaca* L. acts as an adjuvant cardioprotective agent and has anti-oxidative and anti-apoptotic properties (Liu et al., 2009, 2010) and also increases the hypoxia-inducible factor-1α (HIF-1α) in H9c2 cardiac myocytes expression and Akt phosphorylation (Liu et al., 2010) leading to suppression of cardiac cell death. Moreover, leonurine exerts potent cardioprotective effects by either increasing the level of p-PIK, p-AAKT, p-GSK3ß and Bcl-2, or decreasing the levels of caspase 3, cleaved-caspase3 and Bax (Xu et al., 2018). Rosmarinic acid displays effective cardioprotective effects by increasing the enzymes involved in antioxidant activity and regulating the sarcoplasmic reticulum Ca^2+^ homeostasis gene expression (Javidanpour et al., 2017). It also protects against cardiac fibrosis by inhibiting phosphorylation, activating the AMPKα, and nuclear translocation of Smad3 and attenuating cardiac fibrosis by inducing peroxisome proliferator-activated receptors (PPAR-γ) (Lu et al., 2018). Quercetin, found in many plants of Lamiaceae family, has potent cardioprotective effects by inhibiting the secretion of adenosine nucleotide from activated platelets and decreasing the neutrophil function (Kaneider et al., 2004) and reducing the plasma creatine kinase (CK), cardiac TBARS and NO(x) contents (Ahmed et al., 2009; Chiş et al., 2018). Another bioactive compound, viz., Carvacrol, possess cardioprotective activities by suppressing the myocardial ischemic damage and myocardial enzymes, including cardiac troponin T (cTnT), creatine kinase (CK) and lactate dehydrogenase (LDH). It elevates the activities of the antioxidant enzymes superoxide dismutase (SOD), non-enzymatic scavenger glutathione (GSH), and glutathione peroxidase (GSH-PX). It has been shown to inhibit the caspase-3 activation and Bax protein expressions but upregulated Bcl-2 protein expression while reducing the activity of malondialdehyde (Yu et al., 2013). The cardioprotective effects of carvacrol are through the two signalling pathways, viz., MAPK/ERK and Akt/eNOS (Chen Y. et al., 2017). Thymoquinone, one of the active constituents of Thymus plant species, improves cardiac and reduces infarct size and mediate by a decrease in cardiac lactate dehydrogenase and creatine kinase levels and suppressed non-oxidative stress and apoptosis (Xiao et al., 2018). The cardioprotective effect of thymoquinone is by the up-regulating of SIRT1 expression and inhibition of p53 acetylation (Lu et al., 2018). Baicalein, an active component found in *Scutellaria baicalensis* Georgi and *Scutellaria lateriflora* L. and other Lamiaceae plants, exhibits cardioprotective effects besides anti-inflammatory and antioxidant effects. The activation of MAPK and NF-κB pathways in rats is inhibited, decreasing MDA level and increasing the SOD and GSH-Px activity (Kumar et al., 2016; Shi et al., 2018). The compound baicalein, through the Nrf2/Keap1 pathway, protects cardiomyocytes against oxidative stress-induced cells (Cui et al., 2015). There are several other bioactive compounds from plants of Lamiaceae that have similar cardioprotective properties. Polyphenols display antioxidant effects and vasodilatory properties (Portincasa and Calamita, 2019) that reduce the cardiovascular impact. The intake of food rich in flavonoids, and anthocyanidins reduces the death rate due to cardiovascular diseases (Portincasa and Calamita, 2019). With advanced awareness on the benefits of nutraceuticals and plant-derived bioactive molecules for reducing the risk and incidence of CVDs, the demand for herbal formulations rich in antioxidants is increasing (Pandey et al., 2010) for the substitution of synthetic medicines for treating hypertension, hypercholesterolemia and cardiovascular disease. The stability and shelf-life of these bioactive compounds during designing for the prevention of CVDs and tailor-made foods is essential. With the emerging technologies in the last decade, such as high-pressure homogenization, pulsed electric fields, and other non-thermal technologies, gas-plasma were recommended to overcome the nutritional content loss due to the use of severe thermal treatments (Bekut et al., 2018; Do et al., 2018; Patrignani et al., 2021).

### 5.3 Traditional Food Recipe With Lamiaceae

**FIGURE 5:**
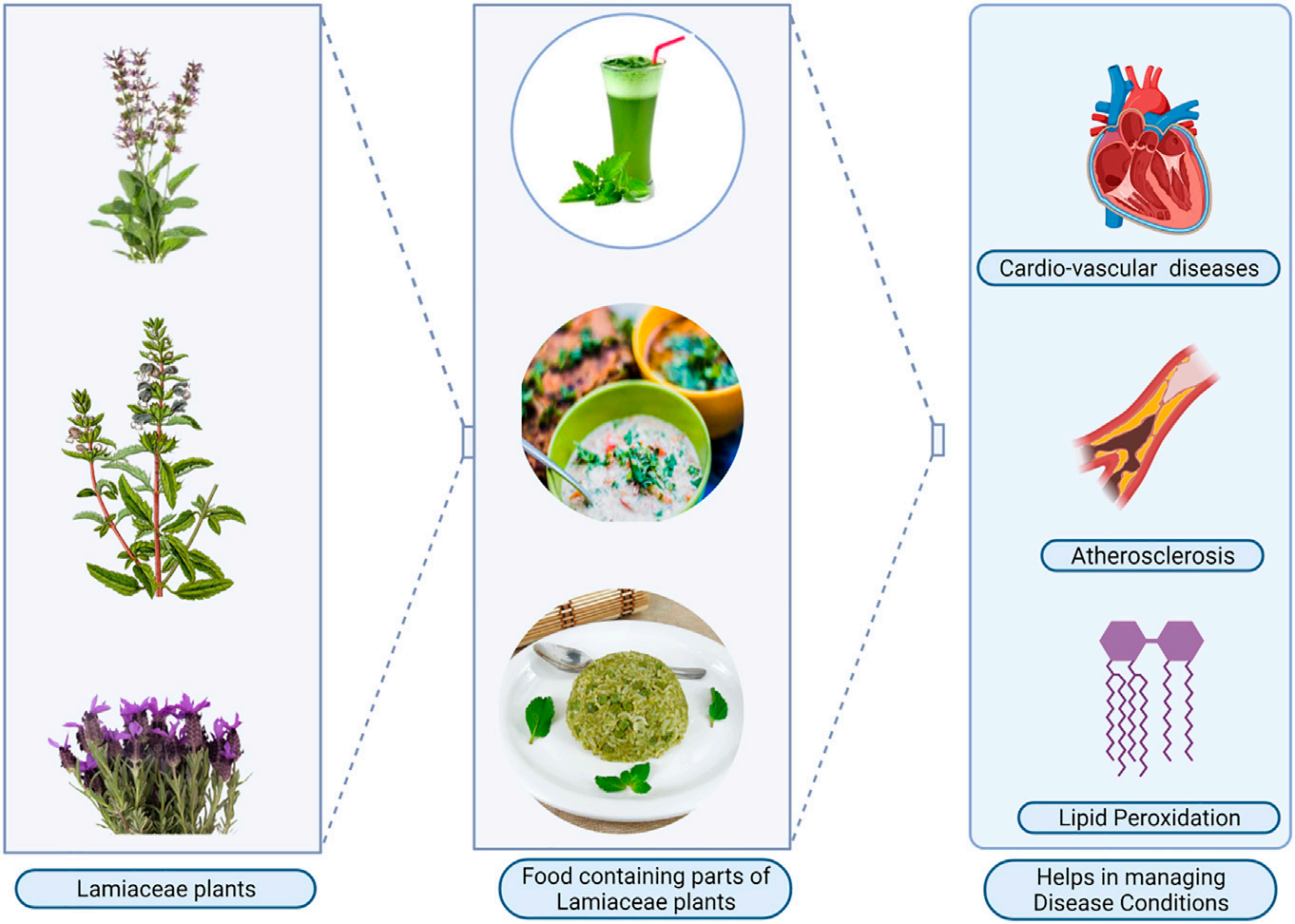
Potential exploration of Lamiaceae as a cardioprotective functional foods (Figure Made in BioRender.com).

The aromatic Lamiaceae plants rich in phytochemicals have high antioxidant activity. The introduction of these phytochemicals into the traditional and modern food recipes has improved the nutritional quality of the foods (Figure 5). Lamiaceae family attains a significantly higher figure for cultural importance. This fact remarks the high significance of the vegetable and herbal tea category in most survey areas (Ali-shtayeh et al., 2008). Lamiaceae are often used in local cuisines to increase the digestibility of cooked food. This confirms that the uses of Lamiaceae plants in food can be relevant to the development of functional foods, pharmafoods or nutraceuticals as they are aromatic and contain many essential oils (da Silva et al., 2021). *Akshomiya* people, during the celebration of the harvest festival—Bihu, collect 101 plant species and prepare vegetable recipes in the evening containing four Lamiaceae species amongst other plants on the first day of the *Bohag Bihu*, also called Goru (cow) Bihu (Gogoi and Zaman, 2013). They believe that this particular recipe has some medicinal values, which is good for health for the next year too. Barros et al. (2011) have reported that several traditional recipes are flavoured by seasoning and to preserve food with the dried leaves and inflorescence of the different species of Lamiaceae plants. These plants are used in conventional delectable soups and summer salads. At the last minute, the fresh leaves of ground ivy are added in different types of soups, viz., soup made of potato, onion and chopped kale, bean soup, and chickpea-based soups. They are also used in stewed beans prepared with vegetables and sausages (Barros et al., 2011). It was reported that the phytochemicals in the refined flour are much lower since the bran and embryo fractions rich in those compounds are removed during the milling process as compared to the whole wheat grains used for the production of the traditional bread (Skendi et al., 2020b). So, the introduction of phytochemicals from the Lamiaceae plants will increase the nutritional quality of the final bread with many health benefits of daily consumption.

Enrichment of bread recipes with essential oils and dry form could inhibit the mycelia growth of *Penicillium* and *Aspergillus* fungi at a concentration range where the bread are considered acceptable based on sensory tests. Moreover, adding aromatic plants in the dry form in the bread recipe is more effective against both fungi studied than essential oils (Skendi et al., 2020a). Extensive studies have been carried out in the Lamiaceae family concerning the essential oils and their use as food preservatives (Debonne et al., 2018b; Císarová et al., 2018) and among them, oregano and thyme are of major importance due to their antimicrobial and antifungal activities (Debonne et al., 2018a; Císarová et al., 2018). Essential oils contain a variety of volatile molecules such as terpenes and terpenoids, phenol-derived aromatic components, and aliphatic compounds (Bakkali et al., 2008; Muslim and Hussin, 2020).

## 6 Novel Nutraceuticals and Medicine From Lamiaceae: Challenges and Opportunities

Many regulations and guidelines exist for the marketing of functional foods and any kind of medications; a number of countries regard nutraceuticals as food supplements and not pharmaceuticals. According to the Food and Drug Administration (FDA) Modernization Act of 1971, pharmaceutical companies and nutraceutical manufacturers should comply with all the guidelines of manufacturing practice, should be responsible for the safety and labeling of the products, and further report in detail if any adverse/toxic effects are associated with the intake of the medicines and nutraceuticals (Dey et al., 2018).

Globally, the consumption of nutraceuticals and plant-based compounds as drug leads has been proliferating due to their alleged efficacy. The evolution of nanoparticle-based mechanisms for drug and formulation the delivery has produced successful results, especially in bioavailability, safety, and stability. However, a lot of challenges exist in this regard. Regulatory bodies are not constituted to monitor the worldwide usage of nutraceuticals and functional foods (Keservani et al., 2017; Helal et al., 2019). The same applies to encapsulation of bioactive compounds in medicines—most countries still have no regulations for risk assessment of bioactive compounds. Because of the lack of consistency, especially in terms of information exchange between different countries, there will be a risk to human health and the environment. This will prevent or limit the marketing of bioactive compounds as therapeutic products (Bazana et al., 2019).

Most importantly, extraction and characterization of the bioactive compounds from their plant sources is a highly time-consuming Moreover, the necessity to obtain a highly purified compound at all times is not always feasible as the process is complicated. Thus, despite all modern developments, isolation and purification of bioactive compounds is a massive challenge (Bucar et al., 2013). Natural products have always been mind-boggling for organic chemists, particularly those involved in the synthesis of new drugs. Though chemical synthesis can suppress or enhance certain activities to obtain or “market” a drug, plant-based drug leads are too complex for this process. Also, syntheses of such molecules are generally not economically viable (Lahlou, 2013).

An exciting aspect of natural compounds is that they do not replicate their natural biological potential *in vitro* or even when synthesized artificially. This can be attributed to the complex metabolic pathways of the living systems that stimulate one compound’s synergistic activity to another. Also, the native conditions of a plant-based compound *in vivo* are difficult to maintain once extracted or even synthesized. As a result, the biological activity of a possible drug lead decreases manifold and natural product chemistry takes a backseat in drug discovery.

Although promising information is available on the efficacy of the plants from Lamiaceae in the treatment of CVDs, the data are too preliminary and mostly fail to explain the exact cellular and molecular mechanisms of action and the respective active compounds. Therefore, future studies should be focused on investigating mechanisms of action, realistic dosages, clinical efficacy, and safety of the extracts and active compounds in the treatment and prevention of cardiac disorders (Uritu et al., 2018). Thus, commercialization of herbal drugs from Lamiaceae, like most other families, is lacking. This review covers a valuable approach that can be adopted to identify new compounds from multiple medicinal plants, which may be effective for treating CVDs.

## 7 Conclusion and Future Prospects

With the rapid spread of several diseases, including COVID-19, there is an urgent requirement to explore and discover new and effective cures for different ailments. Historically, Lamiaceae has a vast tradition of being used as a flavoring spice, for food preservation, and medicinal purposes, with both curative and preventive properties. It is well-known that the members of this family contain a massive array of bioactive compounds—all of which contribute to the overall biological efficacy of the plant extracts, either singly or synergistically (Carović-Stanko et al., 2016). This family needs increased attention from the global scientific community to identify novel bioactive compounds. These bioactive compounds need to be analyzed for their biopharmaceutical potential and focused investigation should be done on their combined, synergistic effect—it is the same way how such compounds function under natural conditions.

Theoretical investigations are already carried out to understand the interactions of the active components with different pathogenic organisms (Haruna and Yahaya, 2021). Therefore, extensive studies (both *in vivo* and *in vitro*) of Lamiaceae, as modulators of physiological responses and biological pathways, are needed to develop enhanced molecules that act against CVDs. The availability of diverse bioactive compounds in Lamiaceae species and their interaction with commercial drugs require further detailed investigation for future pharmaceutical development for herbal medicines. Functional foods and fortified foods are gaining huge importance in the present times due to fast-paced lifestyle, increase in pollution, sedentary habits and others. Beneficial plants like Lamiaceae, when incorporated with everyday foods, can help to enhance their nutritional value manifold.

In countries like Iran, India, South Africa, Pakistan and others, more than 26 species of Lamiaceae are traditionally used for the treatment CVDs and its associated facets like arterial hypertension; such knowledge is also backed by clinical and scientific evidence (Niazi et al., 2019). Therapeutic effects of Lamiaceae on prevention and regulation of blood pressure and heart failure through antioxidant, anti-inflammatory, hypotensive, anti-atherosclerosis, heart rate-regulating, and vasodilating properties re-known in ethnomedicine and nowadays in conventional medicine as well—such studies have even been reported from Morocco (Bouyahya et al., 2017, 2020; Patrignani et al., 2021). With increasing interest in herbs as anti-inflammatory agents in managing chronic inflammation connected to CVDs, research is emerging on the use of herbs in foods. This review is focussed extensively of this aspect—to modulate the lifestyle and food habits of people towards the prevention of CVDs—with different species of Lamiaceae as the focal point.

However, the unavailability or lack of sophisticated analytical tools and knowledge of combinatorial chemistry create a major setback in identifying and characterising bioactive compounds that have a preventive role in CVDs. As such, the development and application of computational tools, docking techniques, models, and other such studies can help to predict and understand the presence of bioactive compounds from Lamiaceae, together with predicting their mechanism of action; in the long run, this might prove to be groundbreaking research. Clinical trials need to be ramped up and ought to be performed with adequate safety measures to demonstrate the effectiveness of medicinal compounds from Lamiaceae so that the human race can opt for plant-based medications in the future, even for life-threatening diseases like CVD. In addition, the lack of knowledge regarding the importance of the members of this valuable family proves to be a limitation. As such, people are clearing away these plants without being able to reap the required benefits from them. Ethnopharmacology should be introduced in the curriculum to educate young minds on the importance of traditional knowledge, ethnic medicines and practices. Even the government and competent authorities should inform people and raise awareness regarding the importance of different species of Lamiaceae; plantation and preservation of more such plants (both in urban and rural areas) can only serve as sources for novel compounds in the future.
