# Supplementary material for: Exploration of Lamiaceae in Cardio Vascular Diseases and Functional Foods: Medicine as Food and Food as Medicine
Source: Front Pharmacol. 2022 Jun 14;13:894814. doi: 10.3389/fphar.2022.894814 (PMC9237463; doi:10.3389/fphar.2022.894814)
Supplement: Supplementary file 2 [file Table3.DOC]

**Supplementary Table 1: Ethno-pharmacological uses and toxicity profiles of some species of Lamiaceae that have a role in the treatment of CVDs.**

| **Sl. No.** | **Plant name** | **Ethno-Pharmacological**  **activity** | **Toxicity** | **References** |
| --- | --- | --- | --- | --- |
|  | *Ajuga iva* (L.) Schreb. | Antidiabetic, anti-hypercholesterolemia, antioxidant/anti-inflammatory, anti-microbial and analgesic | Acute toxicity | (Bouyahya et al., 2020) |
|  | *Ajuga integrifolia* Buch. -Ham. ex D.Don | Antidiabetic | Toxicity not reported | (Rahman et al., 2019) |
|  | *Calamintha vulgaris* (L.) Druce | Antibacterial, Antiproliferative, antioxidant | Low toxicity | (Arantes et al., 2018) |
|  | *Dracocephalum moldavica* L. | Anti-hyperlipidemia, hypolipidemia, hypocholesterolemia, cardioprotective, antihyperlipidemic, diuretic activities and hypolipidemic | Moderate toxicity | (Jiang et al., 2014) |
|  | *Lavandula stoechas* L. | Antioxidant, Anti-inflammatory, Anticonvulsant, sedative, and antispasmodic, Hepatoprotective and hyperglycemia oxidative stress. | Can be toxic for human at certain doses | (Boufellous et al., 2020 ) |
|  | *Ziziphora clinopodioides* Lam. | Antimicrobial, Antifungal, antioxidant and sedative activities | - | (Senejoux et al., 2010) |
|  | *Orthosiphon aristatus* (Blume) Miq. | Antidiabetic, anti-inflammatory, antioxidant, hepatoprotective, analgesic and nephroprotective. | No toxicity of the extract till date | (Yadav et al., 2019; Abdullah et al., 2020) |
|  | *Vitex megapotamica* (Spreng.) Moldenke | Antiatherogenic | No apparent toxicity | (Pires et al., 2018; Brandt et al., 2009) |
|  | *Salvia officinalis* L. | Antioxidant, antimicrobial, Anti-diabetic, Anti-inflammatory, Anti-cancer, Hypo-lipidemic, cognitive, | The extent of cytotoxicity is still unknown | (Sharma et al., 2019) |
|  | *Thymus satureioides* Coss. | Antioxidant, Anti-inflammatory, Antiproliferative, Coagulant | No mortalities | (Ramchoun et al., 2012; Khouya et al., 2015) |
|  | *Salvia miltiorrhiza* Bunge | Antiplatelet and antithrombotic | The extent of cytotoxicity is still unknown | (Fan et al., 2010) |
|  | *Lepechinia caulescens* (Ortega) Epling | Antioxidant and Antidiabetic |  | (Estrada-Soto et al., 2012) |
|  | *Prunella vulgaris* L. | Antioxidant, antithrombotic antihyperlipidemic | No mortalities | (Zargar et al., 2017; Michel et al., 2020) |
|  | *Leucas aspera* (Willd.) Link | Antioxidant activity, Hepatoprotective activity, Anti-inflammatory, Anti-bacterial, Antifungal, Antidiabetic, Antinociceptive, Mosquitocidal, Central Nervous System, Cytotoxicity, Anti-ulcer and antiparkinson’s effect. |  | (Kundu et al., 2018) |
|  | *Leonurus cardiaca* L. | Antimicrobial, antioxidant, anti-inflammatory | The extent of cytotoxicity is still unknown | (Wojtyniak et al., 2013) |
|  | *Sideritis raeseri* Boiss. & Heldr. | Anti-inflammatory, anti-rheumatic, antimicrobial, antinociceptive, anticataract, anti-HIV replication, antifeedant, antiulcer, analgesic, antioxidant. | No mortalities | (Kitic et al., 2012) |
|  | *Satureja cuneifolia*  Ten. | Vasodilation, Anti-hypertensive | The extent of cytotoxicity is still unknown | (Sánchez de Rojas et al., 1999) |
|  | *Teucrium polium* L. | Antidiabetic | Liver toxicity (not conclusive further assessments are needed) | (Albadr et al., 2022) |
|  | *Satureja khuzestanica* Jamzad | Antimicrobial, antioxidant, cytotoxic and apoptotic | Not yet reported | (Soltanzadeh et al., 2018) |

**Supplementary Table 2: Pre-clinical study/ Clinical trial data Clinical data isolated compounds from different species of Lamiaceae.**

| **Sl. No.** | **Bioactive compounds** | **Plant name** | **Pre-clinical study/ Clinical trial data** | **References** |
| --- | --- | --- | --- | --- |
| 01 | Marrubiin | *Leonotis leonurus* [(L.) R.Br.](http://www.theplantlist.org/tpl1.1/record/kew-109449) | Due to the ready availability of marrubiin, high potential applications and ease of modification its contribution to drug discovery and clinical trial thus needs to be put into prospective | (Popoola et al., 2013) |
| 02 | Sinensetin, Eupatorin | *Orthosiphon aristatus* (Blume)  Miq. | To assess its efﬁcacy and safety before submission to clinical studies, more in vivo studies in various animal models including toxicity, pharmacokinetic, pharmacodynamic and bioavailability studies are required. | (Jie et al., 2021) |
| 03 | Eriodictyol | *Satureja cuneifolia* Ten. | The plant extract does not cause any renal toxicity as repeated dosing upon the rat for nine consecutive days. Thus, it requires a clinical study to ensure that its safety profile matches up with the physiology of humans and the long duration that patients usually take on diuretics. | (Michel et al., 2020) |
| 04 | Rosmarinic acid | *Salvia officinalis* L. | Randomized placebo-controlled trial was carried out to study effects on  (a) memory and cognitive functions,  (b) pain, (c) glucose and lipids, (d)  As a widespread metabolite, several traditional plants have been clinically studied using rosmarinic acid as a chemical marker or as an active compound.  Different clinical protocols designed to assess the anti-inflammatory effects and the antioxidant potential of plants containing RA has been employed. | (Ghorbani and Esmaeilizadeh, 2017) |
| 05 | Rosmarinic acid | *Thymus zygis* L. | Numerous clinical trials have been extensively documented to check the side effects related to Non-Steroidal Anti-inflammatory Drugs (NSAIDs). | (Khouya et al., 2016) |
| 06 | Rosmarinic acid, Carvacrol, Camphor,  Caffeic acid, Quercetin, etc | *Thymus satureioides* Coss. | Experimental and clinical studies on atherosclerosis and cancer have also revealed common pathogenic mechanisms that underlie dysfunction of the clotting system.  Comprehensive preclinical and clinical trials on the pharmacological mechanisms of action of *T. satureioides* and its bioactive compounds on molecular targets should be performed. | (Khouya et al., 2019; El Hachlafi et al., 2021) |
| 07 | Salvialonic acid | *Salvia miltiorrhiza* Bunge | Metal ion chelators, such as clioquinol (CQ) and 8-hydroxyquinoline derivatives have been moved into clinical trials.  However long-term use of CQ brings adverse side effects. | (Cao et al., 2013) |
| 08 | Phlomeoic acid | *Phlomoides bracteosa* (Royle ex Benth.) Kamelin and Makhm. | - |  |
| 09 | Luteolin  Linalyl acetate Camphor | *Lavandula angustifolia* Mill. | Clinical trials included in the systematic review ranged from 90 to 160 participants per study. Two studies recruited nulliparous women. Multiparous women were recruited in one study. In two of trials studies the intervention was performed at the beginning of active phase of labor. In another study it was not mentioned clearly. All of studies were conducted in Iran. | (Makvandi et al., 2016) |

**Supplementary Table 3: Natural antioxidants from plants belongs to Lamiaceae with special references to the CVD and functional foods**

| **Sl. No** | **Plant name** | **Responsible antioxidant phytochemicals** | **References** |
| --- | --- | --- | --- |
| 01 | *Satureja hortensis* L. | α-thujene, α-pinene, β-pinene, α-phellandrene, myrcene, α-terpinene, p-cymene, β-phellandrene, γ-terpinene and carvacrol, rosmarinic acid, caffeic acid, naringenin, isoferulic acid and apigenin. Other flavones (luteolin) and their glycosides (apigetrin and vitexin), as well as flavonol (quercetin), flavonol glycosides (isoquercitrin, astragalin, quercitrin) and coumarin derivatives (aesculin and aesculetin). | (Fierascu et al., 2018) |
| 02 | *Ocimum basilicum* L. | linalool, 1,8-cineol, eugenol, methyl cinnamate, camphor, methyl eugenol, methyl chavicol, β-elemene, β-ocimene, camphene, carvacrol, α-bergamotene, α-cadinol and geranial | (Antonescu et al., 2021) |
| 03 | *Lavandula aguistifolia* Mill. | hydroxybenzoic acids (p-hydroxybenzoic acid, protocatechuic acid, vanillic acid, gentisic acid, gallic acid), hydroxycinnamic acids (rosmarinic acid, caffeic acid, p-coumaric, acid, ferulic acid, chlorogenic acid, sinapic acid, cinnamic acid, 4-O-caffeoylquinic, 5-Ocaffeoylquinic) and flavonoids (apigenin and luteolin glycosides, catechin, naringenin, vanillin). New phenolic compounds such as lavandunat, lavandufurandiol, lavandufluoren, lavandupyrones A and B, lavandudiphenyls A and B | (Dobros et al., 2022) |
| 04 | *Leonotis leonurus* [(L.) R.Br.](http://www.theplantlist.org/tpl1.1/record/kew-109449) | Saponins, Flavonoids, Terpenoids, Glycosides, Phenols,Steroids, Alkaloids, Phytosteroids, Phlabotannins, Tannins. | (Mungho et al., 2018) |
| 05 | *Ajuga integrifolia* Buch. -Ham. ex D. Don | phytoecdysones, flavonol glycosides, ergosterol-5,8-endoperoxide, neo-clerodane diterpenoids and iridoid glycosides | (Ullah et al., 2021) |
| 06 | *Clinopodium nepeta* (L.) Kuntze | Gallic acid (GA), rosmarinic acid (RA), caffeine (C) and caffeic acid (CA) | (Khodja et al., 2018) |
| 07 | *Dracocephalum moldavica* L. | Dihydrocaffeic acid derivative, Ferulic acid derivative, Rosmarinic acid, Homoplantaginin, Flavone, Apigenin, | (POVILAITYTé et al., 2001) |
| 08 | *Lagenaria siceraria* (Molina) Standl. | (E)-4-hydroxymethyl-phenyl-6-O-caffeoyl-b-D-glucopyranoside was isolated and identified together with 1-(2-hydroxy-4-hydroxymethyl)-phenyl-6-O-caffeoyl-b-Dgluco-pyranoside, protocatechuic acid, gallic acid, caffeic acid and 3,4-dimethoxy cinnamic acid | (Mohan et al., 2012) |
| 09 | *Lavandula stoechas* L. | flavonoids, catechic tannins, sterols, coumarins, leucoanthocyans and mucilages. Lavandula antineae Maire was found to produce flavonoids and tannins, apigenin 7-glucoside, luteolin, luteolin 7-glucoside, and luteolin 7- glucuronide, flavone glycosides (flavone di-O-glycosides and flavone 7-Omonoglycosides), oleanolic, ursolic and vergatic acids; β-sitosterol; α-amyrin; αamyrin acetate; lupeol; erythrodiol; luteolin; acacetin; vitexin; two longipinane derivatives (longipin-2-ene-7β, 9α-diol-1-one and longipin-2-ene-7β,9α-diol-1-one-9- monoacetate); 7-methoxy coumarin; and lavanol | (Ez zoubi et al., 2020) |
| 10 | *Ocimum gratissimum* L. | Alkaloids, tannins, flavonoids, phytates and oligosaccharides. Eugenol, citral, thymol, linalool, geraniol, and ethyl cinnamate. (Z)-b-ocimene, germacrene D and b-caryophyllene. | (Shah et al., 2018; Oyem et al., 2021) |
| 11 | *Ocimum tenuiflorum* L.  (Syn. *Ocimum sanctum* L.) | β -caryophyllene and eugenol, volatile oils pienene (α- and β -), linalool, carvacrol, camphene, limonene, sabinene, β-elemene, β-caryophyllene, cineole, methyl chavicol, germacrene D, and ursolic acid. | (Shah et al., 2018) |
| 12 | *Ocimum americanum* L. | Citronellal, methyl cinnamate, citronellic acid, eugenol, methyl cinnamate, citronellic acid, eugenol, methyl heptenone, and geraniol, terpinolene, dipentene, crithmene, pinene, caryophyllene, limonene, camphene, sabinene as well as acetic acid in traces  Linalool, eugenol, estragole, camphor, methyl chavicol, methyl cinnamate, β-caryophyllene, trans- α-bergamotene, α-terpineol, germacrene D, thymol, fenchone, p-cymene, and 1,8-cineol as major components. | (Shah et al., 2018) |
| 14 | *Ocimum kilimandscharicum* Gürke | The essential oil profile also reveals camphor, 4-terpineol, limonene, 1,8-Cineole, a-terpineol, linalool, transcaryophyllene, myrtenol, endo-borneol, etc. | (Shah et al., 2018) |
| 15 | *Ocimum campechianum* Mill. | Linalool, elemicin, eugenol, methyleugenol, Linalool/eugenol, 1,8-cineole, thymol/γ-terpinene, and eugenol/β-bisabolene | (Figueiredo et al., 2018) |

**References**

Albadr, Y., Crowe, A., and Caccetta, R. (2022). Teucrium polium: Potential Drug for Type 2 Diabetes Mellitus. *Biology (Basel).* 11, 128. doi:https://doi.org/10.3390/biology11010128.

Antonescu, A. I., Miere, F., Fritea, L., Ganea, M., Zdrinca, M., Dobjanschi, L., et al. (2021). Perspectives on the combined effects of Ocimum basilicum and Trifolium pratense extracts in terms of phytochemical profile and pharmacological effects. *Plants* 10, 1390. doi:https://doi.org/10.3390/plants10071390.

Bouyahya, A., El Omari, N., Elmenyiy, N., Guaouguaou, F.-E., Balahbib, A., El-Shazly, M., et al. (2020). Ethnomedicinal use, phytochemistry, pharmacology, and toxicology of Ajuga iva (L.,) schreb. *J. Ethnopharmacol.* 258, 112875. doi:https://doi.org/10.1016/j.jep.2020.112875.

Cao, Y., Wang, L., Ge, H., Lu, X., Pei, Z., Gu, Q., et al. (2013). Salvianolic acid A , a polyphenolic derivative from Salvia miltiorrhiza bunge , as a multifunctional agent for the treatment of Alzheimer ’ s disease. *Mol. Divesrsity* 17, 515–524. doi:10.1007/s11030-013-9452-z.

Dobros, N., Zawada, K., and Paradowska, K. (2022). Phytochemical Profile and Antioxidant Activity of Lavandula angustifolia and Lavandula x intermedia Cultivars Extracted with Different Methods. *Antioxidants*  11. doi:10.3390/antiox11040711.

El Hachlafi, N., Chebat, A., and Fikri-Benbrahim, K. (2021). Ethnopharmacology, Phytochemistry, and Pharmacological Properties of Thymus satureioides Coss. *Evidence-Based Complement. Altern. Med.* Article ID. doi:https://doi.org/10.1155/2021/6673838.

Estrada-Soto, S., Navarrete-Vázquez. Gabriel, Léon-Rivera, I., Rios, M., Aguilar-Guadarrama, B., Castillo-España, P., et al. (2012). Antihypertensive effect of Lepechinia caulescens extract on spontaneously hypertensive rats. *Phytopharmacology* 2, 170–178.

Ez Zoubi, Y., Bousta, D., Lachkar, M., and Farah, A. (2014). Antioxidant and anti-inflammatory properties of ethanolic extract of Lavandula stocechas L. from taounate region in Morocco. *Int. J. Phytopharm.* 5, 21–26.

Ez zoubi, Y., Farah, A., Zaroual, H., and El Ouali Lalami, A. (2020). Antimicrobial activity of Lavandula stoechas phenolic extracts against pathogenic bacteria isolated from a hospital in Morocco. *Vegetos* 33, 703–711. doi:10.1007/s42535-020-00160-3.

Fan, H. Y., Fu, F. H., Yang, M. Y., Xu, H., Zhang, A. H., and Liu, K. (2010). Antiplatelet and antithrombotic activities of salvianolic acid A. *Thromb. Res.* 126, e17–e22. doi:10.1016/j.thromres.2010.04.006.

Fierascu, I., Dinu-Pirvu, C. E., Fierascu, R. C., Velescu, B. S., Anuta, V., Ortan, A., et al. (2018). Phytochemical profile and biological activities of Satureja hortensis L.: A review of the last decade. *Molecules* 23, 2458.

Figueiredo, P. L. B., Silva, S. G., Nascimento, L. D., Ramos, A. R., Setzer, W. N., da Silva, J. K. R., et al. (2018). Seasonal study of methyleugenol chemotype of Ocimum campechianum essential oil and its fungicidal and antioxidant activities. *Nat. Prod. Commun.* 13, 1055–1058. doi:10.1177/1934578X1801300833.

Ghorbani, A., and Esmaeilizadeh, M. (2017). Pharmacological properties of Salvia officinalis and its components. *J. Tradit. Complement. Med.* 7, 433–440.

Hailu, W., and Engidawork, E. (2014). Evaluation of the diuretic activity of the aqueous and 80% methanol extracts of Ajuga remota Benth (Lamiaceae) leaves in mice. *BMC Complement. Altern. Med.* 14, 1–8.

Jiang, J., Yuan, X., Wang, T., Chen, H., Zhao, H., Yan, X., et al. (2014). Antioxidative and cardioprotective effects of total flavonoids extracted from Dracocephalum moldavica L. against acute ischemia/reperfusion-induced myocardial injury in isolated rat heart. *Cardiovasc. Toxicol.* 14, 74–82. doi:10.1007/s12012-013-9221-3.

Jie, L. H., Jantan, I., Yusoff, S. D., Jalil, J., and Husain, K. (2021). Sinensetin : An Insight on Its Pharmacological Activities , Mechanisms of Action and Toxicity. *Front. Pharmacol.* 11, 1–16. doi:10.3389/fphar.2020.553404.

Khan, S., Khan, T., and Shah, A. J. (2018). Total phenolic and flavonoid contents and antihypertensive effect of the crude extract and fractions of Calamintha vulgaris. *Phytomedicine* 47, 174–183. doi:10.1016/j.phymed.2018.04.046.

Khodja, N. K., Boulekbache, L., Chegdani, F., Dahmani, K., Bennis, F., and Madani, K. (2018). Chemical composition and antioxidant activity of phenolic compounds and essential oils from Calamintha nepeta L. *J. Complement. Integr. Med.* 15. doi:10.1515/jcim-2017-0080.

Khouya, T., Ramchoun, M., Hmidani, A., Amrani, S., Harnafi, H., Benlyas, M., et al. (2015). Anti-inflammatory, anticoagulant and antioxidant effects of aqueous extracts from Moroccan thyme varieties. *Asian Pac. J. Trop. Biomed.* 5, 636–644. doi:10.1016/j.apjtb.2015.05.011.

Khouya, T., Ramchoun, M., Hmidani, A., Amrani, S., Harnafi, H., Benlyas, M., et al. (2016). Chemical characterization and evaluation of antioxidant, anti-inflammatory and anticoagulant activity of aqueous extract and organic fractions of thymus Zygis L. sub SP. Gracilis. *Int. J. Pharm. Sci. Res.* 7, 1396–1405. doi:10.13040/IJPSR.0975-8232.7(4).1396-05.

Khouya, T., Ramchoun, M., Hmidani, A., Amrani, S., Harnafi, H., Benlyas, M., et al. (2019). Acute toxicity and antiproliferative and procoagulant activities of fractions derived from Thymus satureioides of the Moroccan High Atlas. *South African J. Bot.* 121, 568–576.

Kitic, D., Brankovic, S., Radenkovic, M., Savikin, K., Zdunic, G., Kocic, B., et al. (2012). Hypotensive, vasorelaxant and cardiodepressant activities of the ethanol extract of Sideritis raeseri spp. raeseri Boiss & Heldr. *J. Physiol. Pharmacol.* 63, 531–535.

Kundu, S., Salma, U., Sutradhar, M., and Mandal, N. (2018). An Update on the medicinal uses, Phytochemistry and Pharmacology of Leucas Aspera, A medicinally important species. *Int. J. Agric. Innov. Res.* 6, 2319–1473.

Makvandi, S., Mirteimoori, M., Najmabadi, K. M., and Sadeghi, R. (2016). A review of randomized clinical trials on the effect of aromatherapy with lavender on labor pain relief. *Nurse Care Open Acces J.* 1, 42–47. doi:10.15406/ncoaj.2016.01.00014.

Mazimba, O. (2015). Leonotis leonurus : A herbal medicine review. *J. Pharmacogn. Phytochem.* 3, 74–82.

Michel, J., Abd Rani, N. Z., and Husain, K. (2020). A Review on the potential use of medicinal plants From asteraceae and lamiaceae plant family in cardiovascular diseases. *Front. Pharmacol.* 11, 1–26. doi:10.3389/fphar.2020.00852.

Mnonopi, N., Levendal, R.-A., Davies-Coleman, M. T., and Frost, C. L. (2011). The cardioprotective effects of marrubiin, a diterpenoid found in Leonotis leonurus extracts. *J. Ethnopharmacol.* 138, 67–75. doi:https://doi.org/10.1016/j.jep.2011.08.041.

Mohan, R., Birari, R., Karmase, A., Jagtap, S., and Bhutani, K. K. (2012). Antioxidant activity of a new phenolic glycoside from Lagenaria siceraria Stand. fruits. *Food Chem.* 132, 244–251. doi:10.1016/j.foodchem.2011.10.063.

Mungho, T. C., Tobela, G. E., Olasunkanmi, A. O., and Constance, S. R. (2018). Acute toxicity and antihypertensive effects of Artemisia afra and Leonotis leonurus in spontaneously hypertensive rats. *Res. J. Biotechnol.* 13.

Oyem, J. C., Chris-Ozoko, L. E., Enaohwo, M. T., Otabor, F. O., Okudayo, V. A., and Udi, O. A. (2021). Antioxidative properties of Ocimum gratissimum alters Lead acetate induced oxidative damage in lymphoid tissues and hematological parameters of adult Wistar rats. *Toxicol. reports* 8, 215–222. doi:10.1016/j.toxrep.2021.01.003.

Pires, V. A., Cardozo-Junior, E. L., Ortmann, C. F., Maraschin, J. C., Favreto, W. A. J., Donaduzzi, C. M., et al. (2018). Lipid-lowering and antiatherogenic effects of Vitex megapotamica (Spreng.) Moldenke in a mice experimental model. *J. Ethnopharmacol.* 215, 14–20. doi:10.1016/j.jep.2017.12.030.

Popoola, O. K., Elbagory, A. M., Ameer, F., Hussein, A. A., and Africa, S. (2013). Marrubiin. *Molecules* 18, 9049–9060. doi:10.3390/molecules18089049.

POVILAITYTé, V., CUVELIER, M.-E., and BERSET, C. (2001). ANTIOXIDANT PROPERTIES OF MOLDAVIAN DRAGONHEAD (DRACOCEPHALUM MOLDAVICA L.). *J. Food Lipids* 8, 45–64. doi:https://doi.org/10.1111/j.1745-4522.2001.tb00183.x.

Ramchoun, M., Harnafi, H., Alem, C., Büchele, B., Simmet, T., Rouis, M., et al. (2012). Hypolipidemic and antioxidant effect of polyphenol-rich extracts from Moroccan thyme varieties. *ESPEN. J.* 7, 3–8. doi:10.1016/j.clnme.2012.02.005.

Ritter, M., Melichar, K., Strahler, S., Kuchta, K., Schulte, J., Sartiani, L., et al. (2010). Cardiac and electrophysiological effects of primary and refined extracts from Leonurus cardiaca L. (Ph.Eur.). *Planta Med.* 76, E11–E11. doi:10.1055/s-0029-1240698.

Sánchez de Rojas, V. R., Somoza, B., Ortega, T., Villar, A. M., and Tejerina, T. (1999). Vasodilatory effect in rat aorta of eriodictyol obtained from Satureja ohorata. *Planta Med.* 65, 234–238. doi:10.1055/s-1999-13986.

Senejoux, F., Girard, C., Kerram, P., Akber, H., and Berthelot, A. (2010). Mechanisms of vasorelaxation induced by Ziziphora clinopodioides Lam . (Lamiaceae) extract in rat thoracic aorta. *J. Ethnopharmacol.* 132, 268–273. doi:10.1016/j.jep.2010.08.028.

Shah, S., Rastogi, S., and Shasany, A. K. (2018). “Genomic Resources of Ocimum,” in *The Ocimum Genome*, eds. A. K. Shasany and C. Kole (Cham: Springer International Publishing), 99–110. doi:10.1007/978-3-319-97430-9_8.

Sharma, Y., Fagan, J., and Schaefer, J. (2019). Ethno phytochemistry, cultivation and medicinal properties of garden sage (Salvia officinalis L.). *J. Pharmacogn. Phytochem.* 8, 3139–3148.

Soltanzadeh, H., Acik, L., Turk, M., and Houshmand, Massoud Shahsavari, G. (2018). Antimicrobial, antioxidant, cytotoxic and apoptotic activities of satureja khuzestanica. *Gazi Med. J.* 29, 264–270.

Ullah, M. A., Gul, F. Z., Khan, T., Bajwa, M. N., Drouet, S., Tungmunnithum, D., et al. (2021). Differential induction of antioxidant and anti-inflammatory phytochemicals in agitated micro-shoot cultures of Ajuga integrifolia Buch. Ham. ex D.Don with biotic elicitors. *AMB Express* 11, 137. doi:10.1186/s13568-021-01297-3.

Yam, M. F., Shan, C., and Ruan, T. (2018). Vasorelaxant effect of sinensetin via the NO / sGC / cGMP pathway and potassium and calcium channels. *Hypertens. Res.* doi:10.1038/s41440-018-0083-8.

Yam, M. F., Tan, C. S., Ahmad, M., and Shibao, R. (2016). Vasorelaxant action of the chloroform fraction of Orthosiphon stamineus via NO/cGMP pathway , potassium and calcium channels. *Am. J. Chin. Med.* 44, 1413–1439. doi:10.1142/S0192415X16500798.

Zargar, B. A., Ahmad, B., and Bhat, M. F. (2017). Prunella vulgaris Linn . as antihyperlipidemic and antioxidant against high-fat diet induced oxidative stress in wistar rats. *J. Pharmacogn. Phytochem.* 6, 1097–1103.
